# *Lactobacillus rhamnosus* YQ001 binds with GII.4 human noroviruses and inhibits viral replication in zebrafish larvae

**DOI:** 10.1128/aem.01046-25

**Published:** 2025-10-16

**Authors:** Yaqi Yang, Ran An, Xiangjun Zhan, Yunce Liu, Mengge Sun, Shang Chen, Chenang Lyu, Yutong Yang, Qinghua Zhang, Lin Yao, Dapeng Wang

**Affiliations:** 1 Department of Food Science and Technology, School of Agriculture and Biology, Shanghai Jiao Tong University 12474, Shanghai, China; 2 The Key Laboratory of Exploration and Utilization of Aquatic Genetic Resources, Ministry of Education, Shanghai Ocean University 74595, Shanghai, China; 3 The China National Pathogen Collection Center for Aquatic Animals, Shanghai Ocean University 74595, Shanghai, China; 4 Yellow Sea Fisheries Research Institute, Chinese Academy of Fishery Sciences 117919 , Qingdao, China; Anses, Maisons-Alfort Laboratory for Food Safety, Maisons-Alfort, France

**Keywords:** human noroviruses, *Lactobacillus rhamnosus*, zebrafish larvae, antivirals, probiotics, attachment factors

## Abstract

Human noroviruses (HuNoVs) are the primary cause of viral gastroenteritis globally. Nevertheless, available anti-HuNoV approaches remain limited. The current study revealed that a *Lactobacillus rhamnosus* strain, that is, *L. rhamnosus* YQ001, originated de Man, Rogosa and Sharpe (MRS)-based fermentation broth (FB) and cell-free supernatant (CFS) could significantly inhibit the replication of GII.4 HuNoVs in zebrafish larvae (*Danio rerio*), reducing viral RNA titers by approximately 2.18 log_10_ and 1.12 log_10_ copies, respectively. In addition, the inhibitory effect of FB was significantly stronger than that of CFS (*P* < 0.05), while *L. rhamnosus* YQ001 alone demonstrated no inhibitory effect. Zebrafish larvae injected with FB-treated GII.4 HuNoVs demonstrated reduced immune responses (i.e., significantly decreased upregulation of innate immune genes, *ifn* and *mx*, *P* < 0.05), compared to those of larvae injected with untreated GII.4 HuNoVs. Additionally, *in situ* capture RT-qPCR and enzyme-linked immunosorbent assay suggested that membrane proteins, especially C2JVE6, C2JX39 and C2K0J4, in *L. rhamnosus* YQ001, could bind to GII.4 HuNoVs. Altogether, the current study demonstrated the inhibitory effect of *L. rhamnosus* YQ001 originated FB and CFS on GII.4 HuNoVs in zebrafish larvae and identified the binding capacity of membrane proteins from *L. rhamnosus* YQ001. Collectively, these results highlight the synergistic effect of CFS and cell membrane proteins in GII.4 HuNoV control.

**IMPORTANCE:**

Human noroviruses (HuNoVs) are the leading cause of viral gastroenteritis globally, yet effective antiviral treatments remain limited. The current study demonstrated that *Lactobacillus rhamnosus* YQ001 could inhibit GII.4 HuNoV replication in zebrafish larvae. The cell-free supernatant and membrane proteins originated from *L. rhamnosus* YQ001 did work synergistically in GII.4 HuNoVs control. The membrane proteins could bind to the viral capsid. These findings offer a unique insight into the antiviral mechanisms of *L. rhamnosus* YQ001, laying the groundwork for developing probiotic-based foods to anti-HuNoVs.

## INTRODUCTION

Human noroviruses (HuNoVs) are the leading cause of viral gastroenteritis worldwide, posing significant challenges to public health and food safety (1). These highly contagious viruses are responsible for numerous outbreaks, particularly in settings such as restaurants, cruise ships, and healthcare facilities (2). Despite the widespread impact of HuNoVs, effective antiviral treatments remain limited, and there are currently no approved therapeutic agents that directly target HuNoVs (3). This gap in treatment options highlights the urgent need for alternative strategies to control HuNoVs, especially in foodborne contexts where the virus can be readily transmitted through contaminated food and water.

A significant barrier to HuNoV research and drug development is the lack of robust cultivation systems (4). Currently available HuNoV culture systems include human intestinal organoids/enteroids (5) and zebrafish (*Danio rerio*) (6–10). The zebrafish model has emerged as a promising *in vivo* system for studying HuNoVs due to its biological relevance and practical advantages (4). The zebrafish genome is highly homologous to the human genome (11), and zebrafish possess both innate and adaptive immune responses similar to those in humans (12). Notably, human virus infections have been demonstrated in zebrafish larvae, underscoring their value as a model for studying host-pathogen interactions (10, 13, 14). In particular, the zebrafish larva model is a robust and cost-effective model that allows replication and passaging of multiple HuNoV strains (e.g.*,* GII.4, GII.3, and GI.7). This model has been successfully reproduced across laboratories and has been used to investigate antivirals against HuNoVs (15–17).

Interactions between intestinal microbes and viral pathogens play an important role in viral control (18). HuNoVs are known to interact with intestinal microbes, influencing microbial composition, diversity, and gene expression (19). In reverse, some intestinal microbes may have antiviral effects against HuNoVs (20). For instance, transplantation of fecal material into intestinal microbiota-depleted mice resulted in less efficient HuNoV replication (21). Similarly, the inhibitory effects were also observed when *Enterobacter cloacae* was transplanted into gnotobiotic pigs (22). Despite these findings, no specific probiotic strain has been definitively identified as consistently effective against HuNoVs, highlighting a critical gap in understanding the antiviral potential of intestinal microbes.

In this study, we utilized the zebrafish larva model to investigate the antiviral potential of *Lactobacillus rhamnosus* YQ001 against GII.4 HuNoVs, one of the most prevalent strains responsible for gastroenteritis outbreaks (23). The findings of the current study will provide compelling evidence for the antiviral properties of *L. rhamnosus* YQ001 originated fermentation broth (FB) and cell-free supernatant (CFS) in controlling HuNoV replication.

## RESULTS

### *L. rhamnosus* YQ001 originated CFS, and bacterial cells act synergistically in the inhibition of GII.4 HuNoV replication in zebrafish larvae

Zebrafish larvae are known as a robust HuNoV replication model (6). In the current study, we observed a peak in GII.4[P31] HuNoVs amount at 2 days post-injection (dpi), with viral RNA levels increasing by approximately 2.88 log_10_ copies (Fig. S1). Based on these findings, 2 dpi was chosen in the following studies for sample collection.

**Fig 1:**
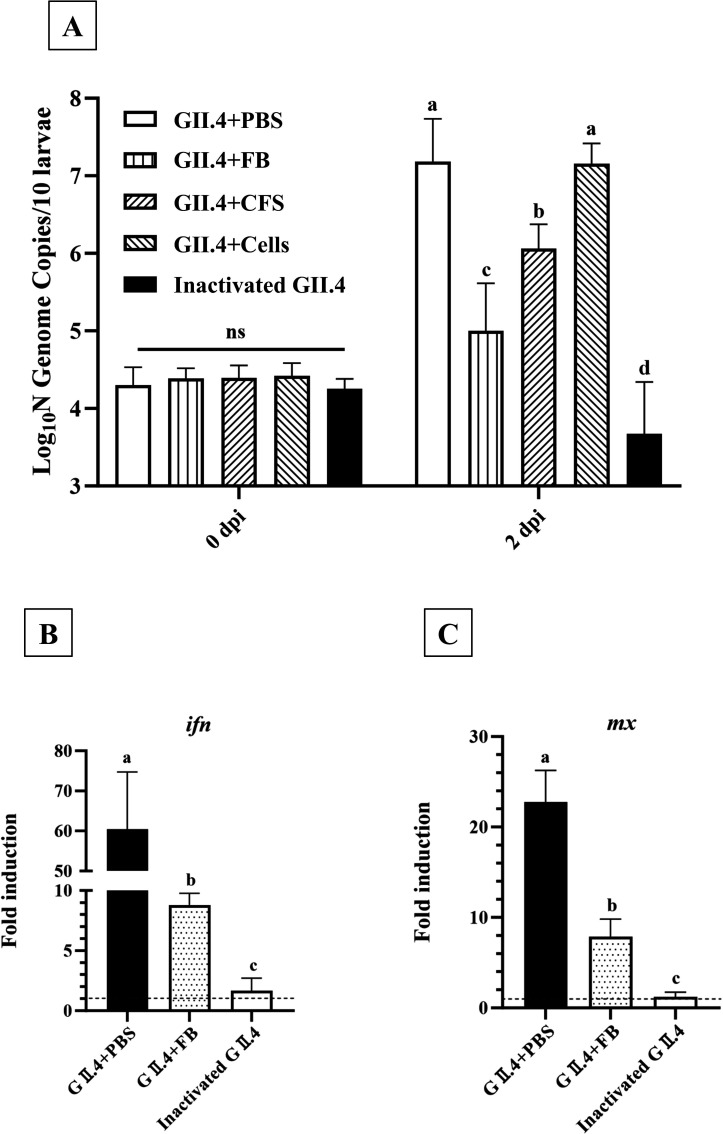
Evaluation of antiviral activity of *L. rhamnosus* YQ001 against GII.4 HuNoV replication. (**A**) Detection of the antiviral effects of *L. rhamnosus* YQ001 against GII.4 HuNoVs. Zebrafish larvae were infected with GII.4 HuNoVs mixed with (i) phosphate-buffered saline (PBS), (ii) *L. rhamnosus* YQ001 fermentation broth (FB), (iii) *L. rhamnosus* YQ001 cell-free supernatant (CFS), or (iv) *L. rhamnosus* YQ001 cells. In the control group (v), larvae were infected with inactivated GII.4 HuNoVs. Bars represent viral RNA levels (five independent experiments), quantified by RT-qPCR. dpi, days post-injection. (**B and C**) The effect of GII.4 HuNoVs pre-incubated with FB on the expression of *ifn* and *mx*, determined by RT-qPCR. Bars represent the fold-induction in zebrafish larvae injected with GII.4 HuNoVs, FB pre-incubated GII.4 HuNoVs, and inactivated GII.4 HuNoVs compared to zebrafish larvae injected with PBS and normalized to the housekeeping genes (three independent experiments). For all graphs, groups of 10 zebrafish larvae were collected at each time point for each independent experiment. Mean values ± standard deviation are presented. Different lowercase letters above the bars indicate statistical significance between groups, and ns indicates no statistical difference between groups, as determined by one-way ANOVA test (*P* < 0.05).

To investigate the antiviral potential of *L. rhamnosus* YQ001 against GII.4 HuNoVs, zebrafish larvae were infected with: (i) mixture of phosphate-buffered saline (PBS) and GII.4 HuNoVs, (ii) mixture of *L. rhamnosus* YQ001 FB and GII.4 HuNoVs, (iii) mixture of *L. rhamnosus* YQ001 CFS and GII.4 HuNoVs, (iv) mixture of *L. rhamnosus* YQ001 cells and GII.4 HuNoVs, and (v) inactivated GII.4 HuNoVs (Fig. 1A). No visible disease symptoms or significant effects on survival were observed in infected larvae after microinjection, with an average post-injection survival percentage exceeding 85% (Fig. S2).

Although the bacterial cells alone did not exhibit the inhibitory effect, both *L. rhamnosus* YQ001 originated FB and CFS inhibited the viral replication significantly (*P* < 0.05) (Fig. 1A). In addition, the decreased replication resulted in a corresponding decrease in related immune genes upregulation (Fig. 1B and C). Specifically, at 2 dpi, injection of GII.4 HuNoVs led to a significant upregulation of *ifn* and *mx* expression, with an average increase of 60-fold and 23-fold, respectively, compared to PBS-injected larvae (Fig. 1B and C). However, when GII.4 HuNoVs were pre-incubated with *L. rhamnosus* YQ001 originated FB, the upregulation of *ifn* and *mx* was limited to ~9-fold and ~8-fold, respectively. Inactivated GII.4 HuNoV suspension was included as a control and did not trigger upregulation of *ifn* and *mx* (Fig. 1B and C). Moreover, larvae treated with FB alone exhibited only minor changes in the expression of *ifn* and *mx*, indicating that the reduced expression of immune genes in the FB pre-incubated GII.4 HuNoV group was primarily a result of lower viral replication (Fig. S3).

**Fig 2:**
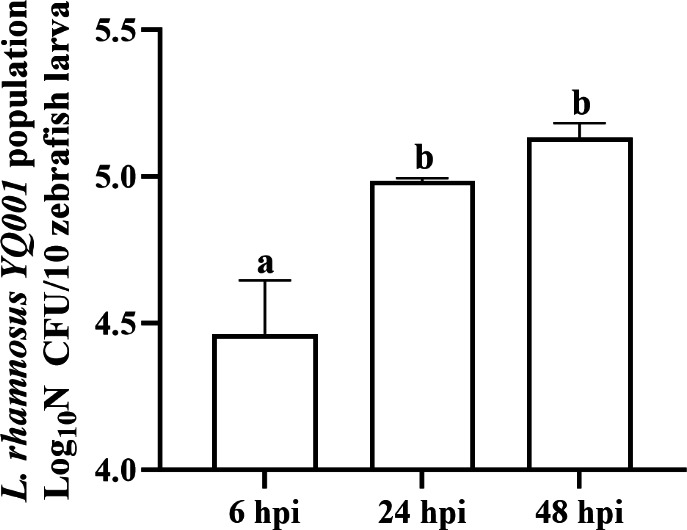
*L. rhamnosus* YQ001 population detected in zebrafish larvae at different time points. Groups of 10 zebrafish larvae were collected at each time point for each independent experiment. Mean values ± standard deviation are presented (three independent experiments). Different lowercase letters above the bars indicate statistical significance between groups, as determined by one-way ANOVA test (*P* < 0.05). hpi, hours post-injection.

To evaluate the persistence and potential proliferation of *L. rhamnosus* YQ001 within the larvae, the bacterial population in FB-injected larvae was measured at 6, 24, and 48 h post-injection (hpi). The bacterial population significantly increased from 6 hpi (4.46 ± 0.18 log_10_ CFU per 10 larvae) to 24 hpi (4.99 ± 0.01 log_10_ CFU per 10 larvae) (*P* < 0.05) and stabilized at 48 hpi (5.13 ± 0.05 log_10_ CFU per 10 larvae) (Fig. 2). Overall, the bacterial population increased by approximately 0.67 log₁₀ CFU per 10 larvae, indicating that *L. rhamnosus* YQ001 remained relatively stable within the yolk without undergoing uncontrolled proliferation (Fig. 2).

**Fig 3:**
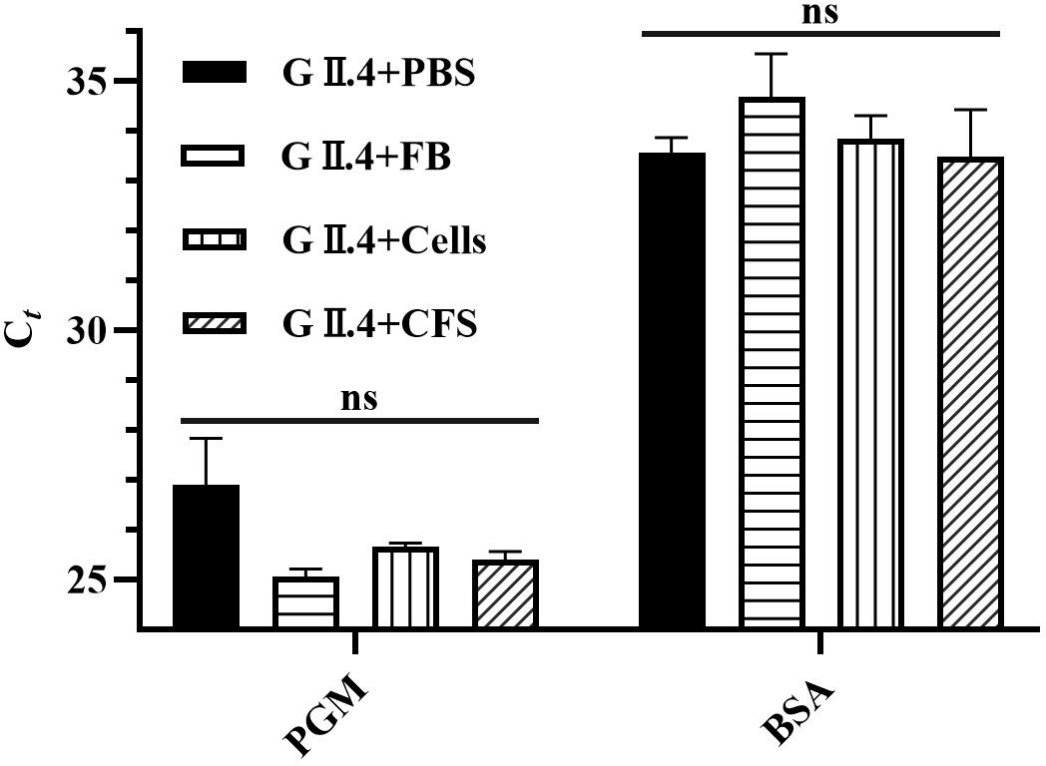
Evaluation of GII.4 HuNoV binding to PGM in the presence of *L. rhamnosus* YQ001-derived FB, CFS, and cells by *ISC*-RT-qPCR. Mean values ± standard deviation are presented (three independent experiments). ns indicates no statistical difference between groups.

Moreover, we employed *in situ* capture RT-qPCR (*ISC*-RT-qPCR) to distinguish intact HuNoVs by capturing intact virus particles using porcine gastric mucin (PGM) (24). The *C_t_* value of virus particles treated with the FB, CFS, or cells of *L. rhamnosus* YQ001 showed no significant difference compared to untreated viral particles (*P* < 0.05), indicating that no significant reduction in virus binding to PGM was observed (Fig. 3). This suggested that the antiviral component of *L. rhamnosus* YQ001 did not severely disrupt the capsid structure of GII.4 HuNoVs.

Of note, although the bacterial cells alone did not exhibit any antiviral effect, *L. rhamnosus* YQ001 originated FB reduced viral titers by about 2.18 log_10_ copies, while the CFS reduced them by approximately 1.12 log_10_ copies (Fig. 1A). In other words, *L. rhamnosus* YQ001 originated FB showed superior inhibitory effects on viral replication compared to the CFS. Given that the primary difference between FB and CFS is the presence of bacterial cells, we hypothesized that these cells might play a supporting role in inhibiting viral replication. A previous study has shown that certain probiotic bacteria could bind to HuNoV P protein and inhibit its attachment to HT-29 cells (25). Based on this, we hypothesized that the cells of *L. rhamnosus* YQ001 might have a similar binding capability, potentially synergizing with CFS to inhibit GII.4 HuNoV replication.

### Identification of *L. rhamnosus* YQ001 originated membrane proteins which could bind with GII.4 HuNoVs

**Fig 4:**
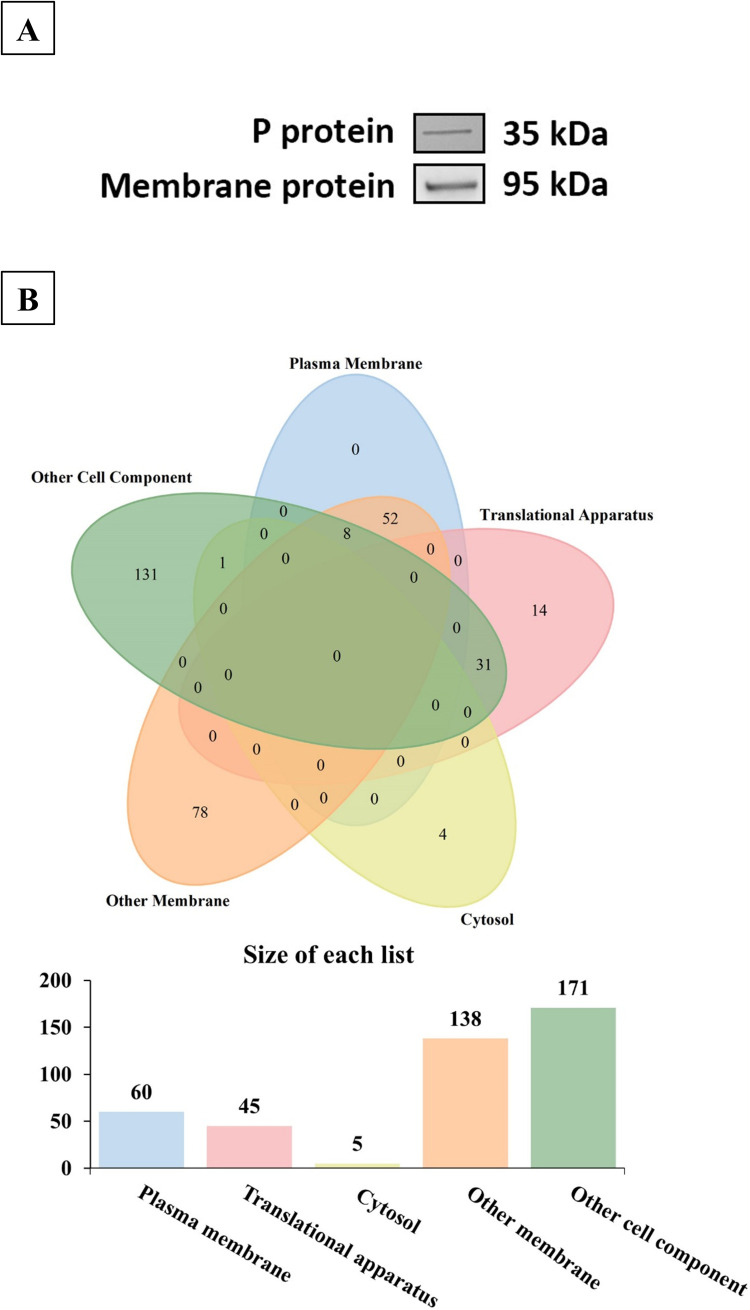
Binding of GII.4 HuNoV P protein to *L. rhamnosus* YQ001 originated membrane proteins. (**A**) *L. rhamnosus* YQ001 cells were incubated with GII.4 P protein at 37°C for 1 h. After washing, the bacterial samples were analyzed by Western blot using anti-HuNoV GII.4 monoclonal antibodies. P protein signals (~35 kDa) indicate binding to the bacterial cell surface. Bacterial membrane proteins were extracted, separated by SDS-PAGE, and transferred to PVDF membranes. The blots were incubated with GII.4 P protein at 4°C overnight, and binding was detected with anti-HuNoV GII.4 antibodies. A clear band at ~95 kDa suggests binding of the P protein to membrane protein(s) of similar molecular weight. (**B**) Cellular sublocalization of potential attachment factors of GII.4 HuNoVs in *L. rhamnosus* YQ001 identified by mass spectrometry. Membrane proteins (~95 kDa band) interacting with the P protein were excised and digested from PVDF membranes, followed by LC-MS/MS analysis. Subcellular localization of identified proteins was annotated based on UniProt database entries.

To investigate the role of *L. rhamnosus* YQ001 cells in the previous synergistic inhibitory effect, we cloned the P protein (35 kDa) of GII.4 HuNoVs as a model for interactions with the bacterial cells. After incubation of the GII.4 P protein with *L. rhamnosus* YQ001, a distinct band at approximately 35 kDa was detected by Western blot using anti-HuNoV GII.4 antibodies, which confirmed that *L. rhamnosus* YQ001 cells could bind to the GII.4 HuNoV P protein (Fig. 4A).

Next to that, we extracted bacterial membrane proteins from *L. rhamnosus* YQ001 and conducted a virus overlay assay with the GII.4 P protein (26) and found that membrane proteins (~95 kDa) of *L. rhamnosus* YQ001 banded with the P protein, as shown in Fig. 4A. These findings suggested the presence of proteinaceous attachment factors for GII.4 HuNoVs, approximately 95 kDa in size, within the membrane proteins of *L. rhamnosus* YQ001.

To further investigate proteinaceous attachment factors from *L. rhamnosus* YQ001, membrane proteins of approximately 95 kDa in size from *L. rhamnosus* YQ001 were isolated and subjected to identification. Using an Orbitrap Exploris 480 mass spectrometer, a total of 319 proteins were successfully identified. To narrow down the candidates, we performed a subcellular-localization prediction of proteins and retained membrane proteins (*n* = 60) (Fig. 4B). Considering the potential degradation of proteins during electrophoresis and the known deviations between SDS-PAGE-indicated molecular weights and actual protein masses, we selected 16 membrane proteins with predicted molecular weights ranging from 70 to 110 kDa for further analysis (Table 1).

**TABLE 1:** Details of potential attachment factors from *L. rhamnosus* YQ001 of GII.4 HuNoVs

Uniprot ID	Description	No. of specific peptides	Molecular weight (kDa)
C2JY75	Copper-exporting ATPase	11	79.5
C2JVE6	ATP-dependent zinc metalloprotease	46	78.2
C2JX39	DNA topoisomerase 4 subunit A	7	90.7
C2JVR3	Protein translocase subunit SecA	46	89.4
C2JXY1	Penicillin-binding protein, transpeptidase domain protein	43	77.4
C2JWP5	Penicillin-binding protein, transpeptidase domain protein	32	76.8
C2JT23	Arylsulfatase	24	78.7
C2JWA3	Arylsulfatase	24	84.0
C2JTK1	Cyclic-di-AMP phosphodiesterase	21	74.7
C2K0J4	Penicillin-binding protein, transpeptidase domain protein	6	70.8
C2K153	Phosphotransferase system, EIIC	1	70.3
C2K1E4	DNA topoisomerase 4 subunit A	46	77.7
C2JUC2	Protein translocase subunit SecA	3	71.4
C2JWZ7	Phosphoenolpyruvate-dependent sugar phosphotransferase system, EIIA 2	11	70.0
C2JZ39	Magnesium-transporting ATPase, P-type 1	11	96.4
C2JYD6	ABC transporter, ATP-binding protein	25	87.3

**Fig 5:**
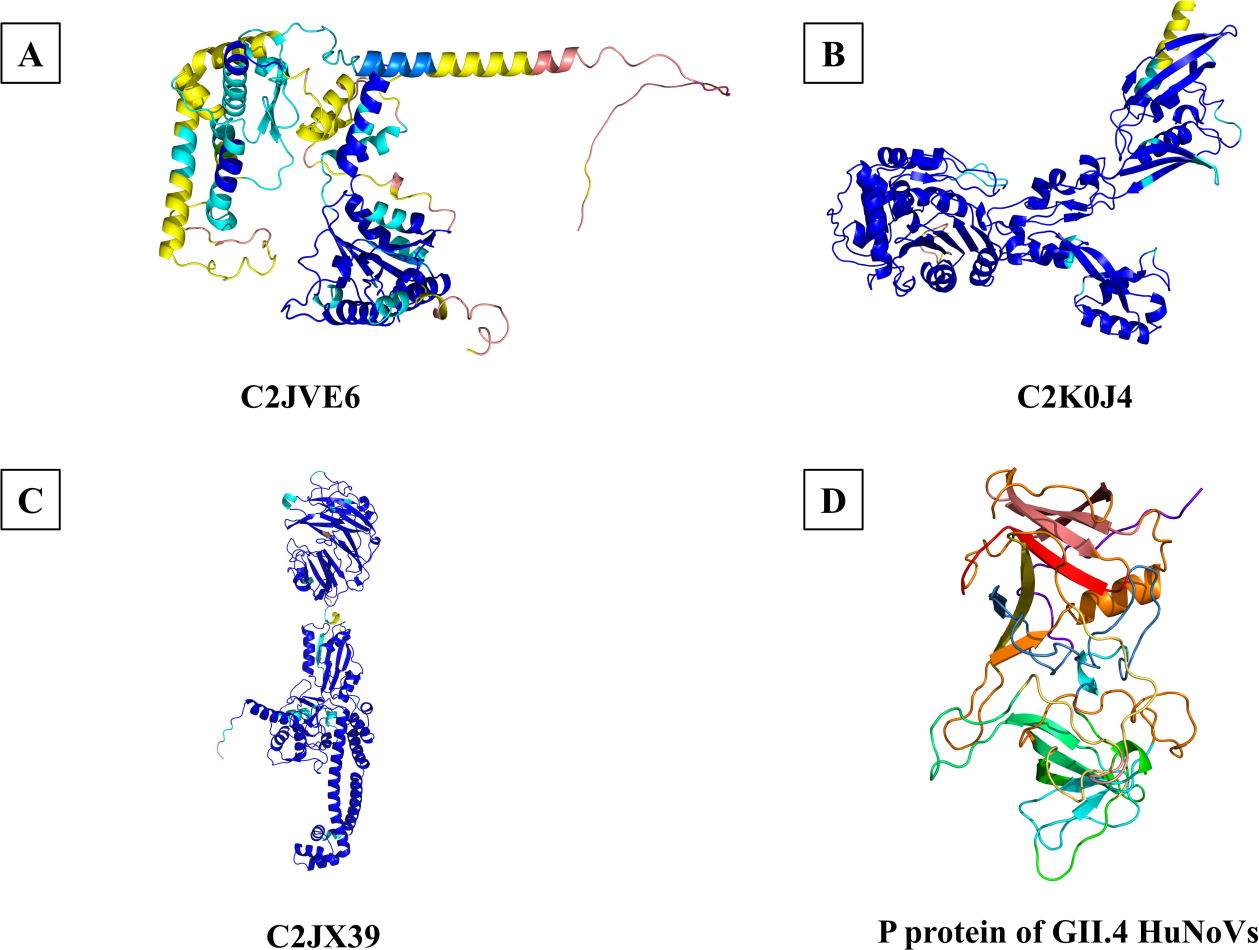
Three-dimensional structure models of C2JVE6, C2K0J4, C2JX39, and GII.4 HuNoV P protein. (**A–C**) Three-dimensional structure models of C2JVE6, C2K0J4, and C2JX39 obtained from the AlphaFold Protein Structure Database via their respective UniProt entries. (**D**) GII.4 HuNoV P protein conformation downloaded from the PDB database.

**Fig 6:**
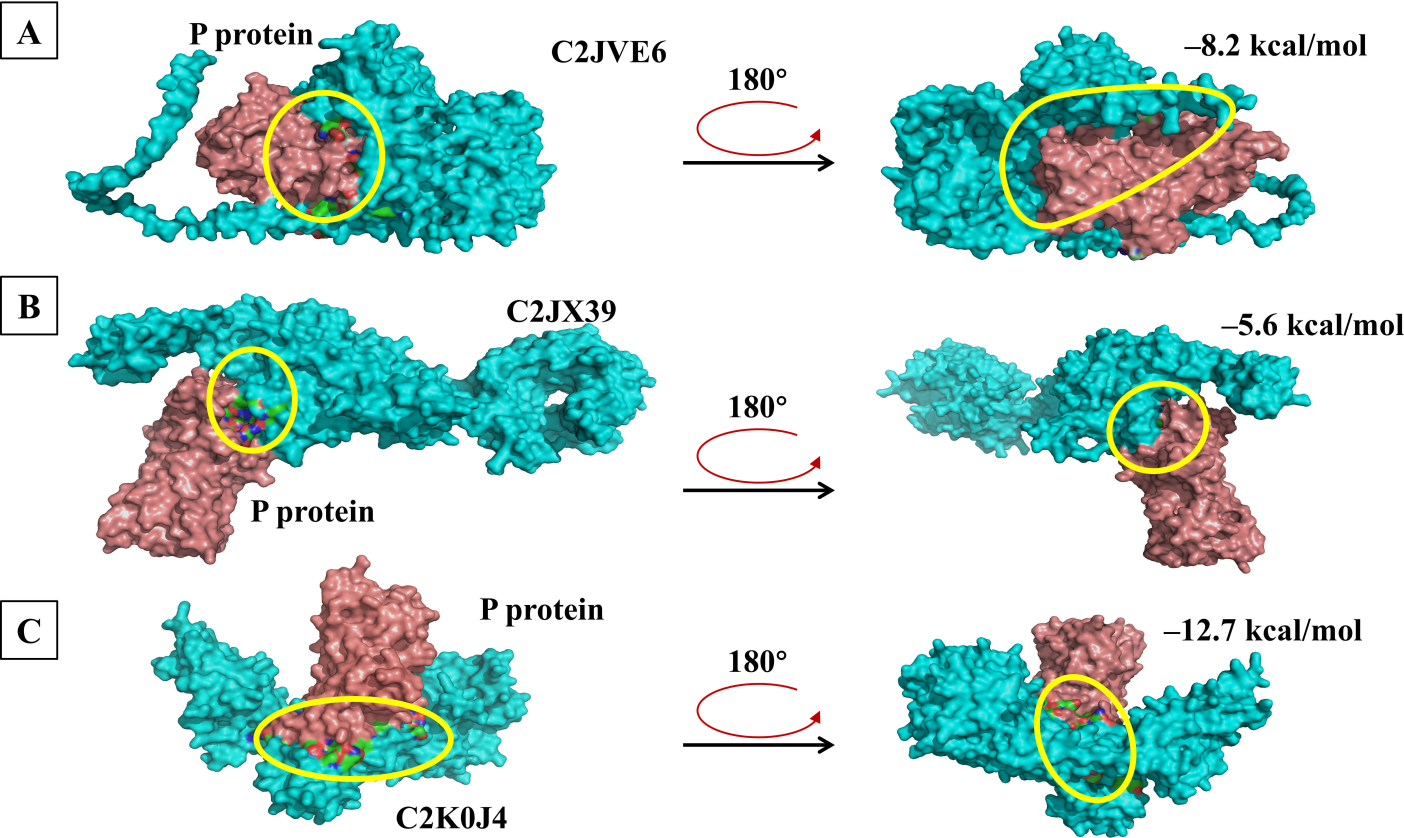
Molecular docking of GII.4 HuNoV P protein with C2JVE6, C2JX39, and C2K0J4. (**A–C**) Molecular docking results of GII.4 HuNoV P protein with C2JVE6, C2JX39, and C2K0J4, along with the corresponding docking energies (Δ^i^*G*). The right side of each image shows the left image rotated 180° horizontally. The interaction interfaces are indicated with solid circles. Detailed interaction forces between the P protein and each candidate protein are shown in Fig. S4 through S6 and Table S1.

**Fig 7:**
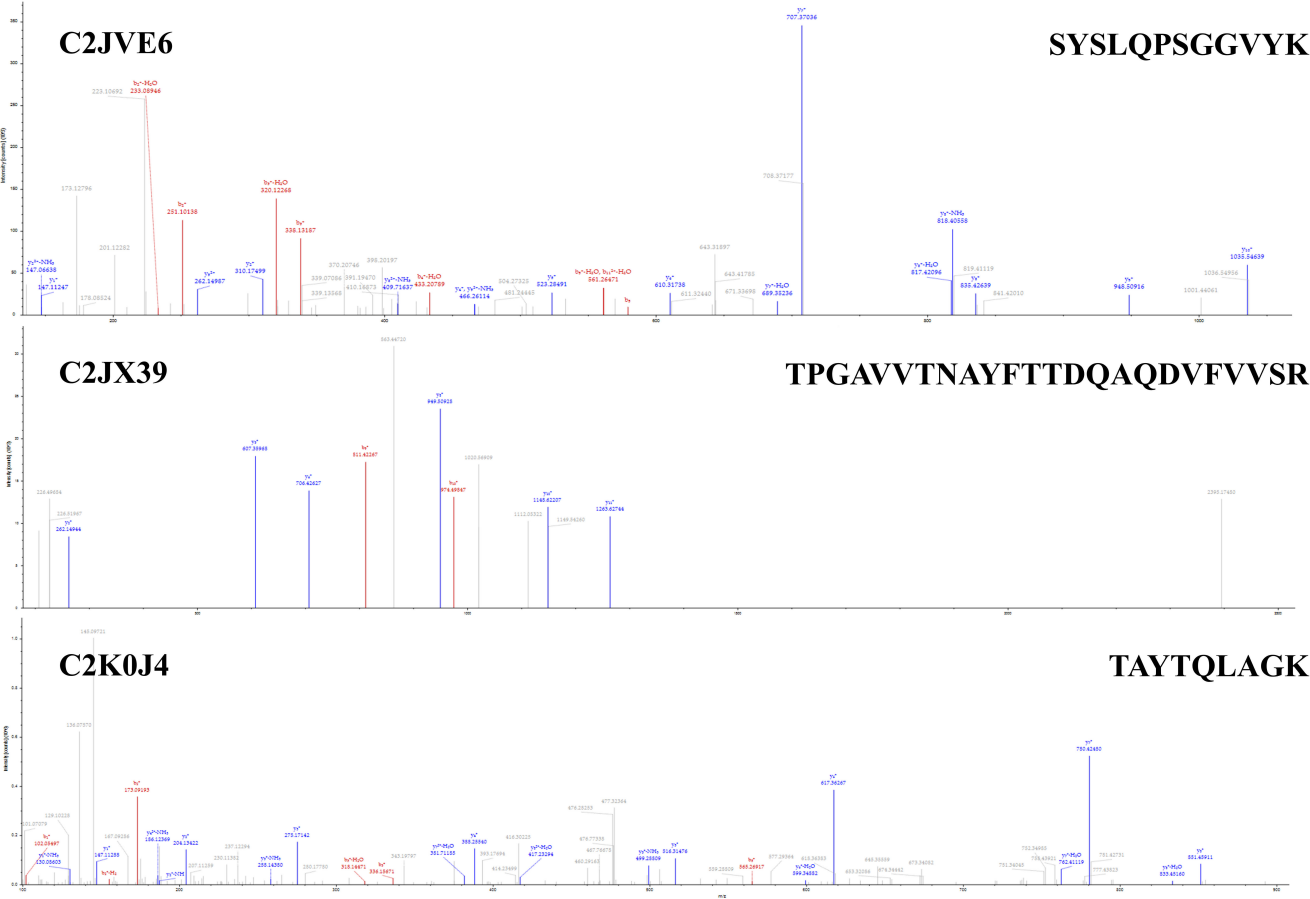
Tandem MS spectra of unique peptides of C2JVE6, C2JX39, and C2K0J4. Membrane proteins from *L. rhamnosus* YQ001 were separated by SDS-PAGE, transferred to PVDF membranes, and detected by Western blot. Target bands were excised and subjected to trypsin digestion. Peptides were analyzed using an Orbitrap Exploris 480 mass spectrometer in full scan/ddMS^2^ mode (*m/z* 350–1,500, resolution 60,000 for MS1 and 15,000 for MS/MS, HCD collision energy 30%). The identified peptides are marked on the top right of each MS spectrum. The corresponding protein is shown in the top left corner.

Molecular docking was performed between each of the 16 candidate membrane proteins and GII.4 HuNoV P protein using GRAMM-X and AlphaFold 3. Based on the docking results, we prioritized candidate proteins that exhibited low predicted docking energies (Δ^i^*G*) and interaction interfaces located primarily within the P2 subdomain of the GII.4 P protein, a region known to be a critical site for virus-host interactions (27). Furthermore, proteins whose predicted docking sites were located primarily in extracellular regions were considered more likely to serve as attachment factors. According to these criteria, C2JVE6, C2JX39, and C2K0J4 from *L. rhamnosus* YQ001 were selected for further study. The structure models and molecular docking results of the candidate membrane proteins and the GII.4 HuNoV P protein are shown in Fig. 5 and 6. C2JVE6, C2JX39, and C2K0J4 showed interface areas of 2,227.4, 1,000.3, and 2,380.8 Å², with Δ^i^*G* of −8.2, −5.6, and −12.7 kcal/mol, respectively (Fig. 6). Detailed descriptions of the interaction forces and binding bonds are available in Table S1 and Fig. S4 through S6. The unique peptides of C2JVE6, C2JX 39, and C2K0J4 were identified and confirmed by mass spectrometry analysis, as shown in Fig. 7. The presence of these unique peptides indicated the existence of the corresponding proteins.

### Binding affinity of identified proteins with GII.4 HuNoV and P protein

**Fig 8:**
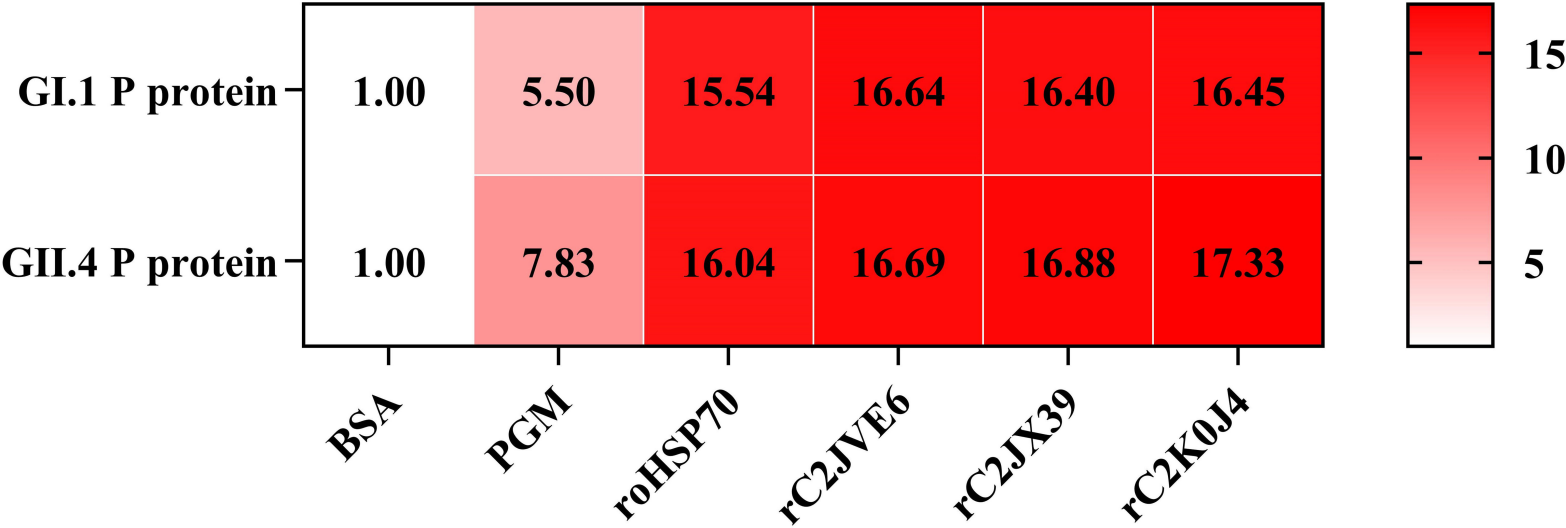
The binding ability of BSA, PGM, roHSP70, rC2JVE6, rC2JX39, and rC2K0J4 to GI.1 and GII.4 P proteins. A heat scale for the S/N ratio is shown on the right, with higher ratios represented by darker red. Specific S/N ratios are displayed in the corresponding boxes, reflecting the binding affinities of the tested samples to GI.1 and GII.4 P proteins. BSA, bovine serum albumin, used as a negative control; PGM, porcine gastric mucin, a known positive control for GI.1 and GII.4 P protein binding; roHSP70, recombinant oyster heat shock protein 70, a reported attachment factor for GI.1 and GII.4 P proteins; S/N, signal/negative signal.

The binding ability of recombinant C2JVE6/C2JX39/C2K0J4 (rC2JVE6/rC2JX39/rC2K0J4) to GII.4 HuNoV P protein was evaluated by an enzyme-linked immunosorbent assay (ELISA) (Fig. 8). These proteins demonstrated strong binding ability to GII.4 P protein, with signal/negative signal (S/N) ratios of 16.69 (rC2JVE6), 16.88 (rC2JX39), and 17.33 (rC2K0J4). These values were comparable to the positive control recombinant oyster heat shock protein 70 (roHSP70) (16.04) and significantly higher than PGM (7.83) (*P* < 0.05), highlighting the strong binding affinity of selected attachment factors to HuNoVs (Fig. 8). Given the genetic diversity among HuNoV genogroups (28), we further evaluated the cross-genogroup binding potential of these proteins using the GI.1 P protein. Remarkably, rC2JVE6, rC2JX39, and rC2K0J4 also showed strong binding affinity to GI.1 HuNoV P protein, with S/N ratios of 16.64 (rC2JVE6), 16.40 (rC2JX39), and 16.45 (rC2K0J4), comparable to roHSP70 (15.54) and significantly higher than PGM (5.50) (*P* < 0.05) (Fig. 8). Notably, these identified proteins showed no statistically significant difference in binding affinity between GII.4 and GI.1 P proteins (Fig. 8), suggesting that rC2JVE6, rC2JX39, and rC2K0J4 could bind to different HuNoV genogroups.

*ISC*-RT-qPCR further confirmed the binding ability of rC2JVE6/rC2JX39/rC2K0J4 to intact viral particles from different clinical samples (Table 2). For the GII.4-Sydney strain, their binding efficiencies were comparable to those of the positive controls PGM and roHSP70, with no significant differences observed (*P* > 0.05). In contrast, for GII.4[P31] and GII.4[P16], slight but significant differences were observed (*P* < 0.05).

**TABLE 2:** GII.4 HuNoV capture ability of attachment factors^*a*^

Simple no.	Virus	PGM	roHSP70	rC2JVE6	rC2JX39	rC2K0J4	BSA
1	GII.4-Sydney	4.78 ± 0.55^a^	4.64 ± 0.05^a^	4.6 ± 0.07^a^	4.32 ± 0.18^a^	4.44 ± 0.20^a^	N/A
2	GII.4[P31]	5.45 ± 0.18^a^	5.45 ± 0.18^ab^	4.46 ± 0.29^cd^	3.86 ± 0.09 ^d^	4.68 ± 0.47^bc^	N/A
3	GII.4[P16]	5.03 ± 0.28^b^	5.75 ± 0.12^a^	4.56 ± 0.11^c^	4.37 ± 0.28^c^	4.36 ± 0.15^c^	N/A

^*a*^ Mean values ± standard deviation are presented. Different lowercase letters indicate statistical significance between groups, as determined by one-way ANOVA test (*P *< 0.05). Data are expressed as log_10_ copies/mL. N/A, not applicable; C*_t_* values measured by *ISC*-RT-qPCR were greater than 40, indicating undetectable viral RNA levels.

Nevertheless, these findings confirmed the capacity of C2JVE6, C2JX39, and C2K0J4 to bind intact GII.4 HuNoVs and highlighted their roles as attachment factors in inhibiting GII.4 HuNoV replication by *L. rhamnosus* YQ001 originated FB. 

## DISCUSSION

The current study reveals that *L. rhamnosus* YQ001 exhibits potent antiviral activity against GII.4 HuNoVs. Using zebrafish larvae as a replication model, we demonstrated that FB and CFS derived from de Man, Rogosa and Sharpe (MRS)-based *L. rhamnosus* YQ001 cultures significantly inhibited viral replication. FB showed superior antiviral efficacy, reducing viral RNA titers by approximately 2.18 log₁₀ copies, whereas CFS resulted in a reduction of about 1.12 log₁₀ copies. Additionally, by reducing viral replication, FB lowered the overall viral load, which in turn reduced the upregulation of virus-induced immune genes (*ifn* and *mx*). Proteomic and molecular docking analyses identified three membrane proteins, namely C2JVE6, C2JX39, and C2K0J4, as key viral attachment factors that exhibited strong binding affinity to HuNoV capsid proteins. These findings underscore the dual benefits of *L. rhamnosus* YQ001 FB, including direct inhibition of viral replication and physical binding of viral particles, highlighting its potential as a microbe-based antiviral agent.

To date, the efficacy of potential antiviral probiotics against HuNoV infections remains varied. A case-controlled study found that *L. casei* strain Shirota in probiotic-fermented milk reduced fever duration in elderly patients with HuNoV gastroenteritis (29). However, other double-blind, placebo-controlled trials on probiotics such as *L. acidophilus* in children with acute viral gastroenteritis, including HuNoVs, found no significant impact on reducing diarrhea duration or viral clearance (30, 31). Due to limitations in HuNoV cultivation system, murine noroviruses (MNVs) are commonly used as a surrogate to assess the antiviral effects of probiotics. Some lactic acid bacteria isolated from fermented foods and fecal samples could restrict MNV replication (32, 33). Notably, *Limosilactobacillus fermentum* PV22 exhibited strong anti-MNVs effects, with the *gadB* gene linked to antiviral activity through γ-aminobutyric acid production (33). Animal models, including gnotobiotic pigs, chimpanzees, and murine models, have been used to study HuNoV infection. Nevertheless, using these animal models in high-throughput antiviral screening is challenging (34). Zebrafish have emerged as a viable model for *in vivo* antiviral assessment. Studies have shown that compounds such as fucoidan and 2′-C-methylcytidine could inhibit HuNoV replication in zebrafish larvae (6, 16). Our findings with *L. rhamnosus* YQ001 in the zebrafish model underscore the potential of microbial treatments for HuNoV inhibition and confirm the method for assessing anti-HuNoV efficacy of microbes *in vivo* (35).

HuNoV infection in zebrafish larvae triggers an IFN-mediated innate immune response (6, 14). We observed a significant upregulation of *ifn* (~60-fold) and *mx* (~23-fold) after the infection. This activation pattern closely resembled the immune responses observed in HuNoV-infected calves and murine models (36, 37). When GII.4 HuNoVs were pre-incubated with *L. rhamnosus* YQ001 originated FB, the upregulation of *ifn* and *mx* was limited to ~9-fold and ~8-fold, respectively (Fig. 1B and C), which corresponds to the lower viral load in this group (5.00 ± 0.35 log_10_ copies/10 larvae) compared to the GII.4 HuNoV-infected group (7.18 ± 0.27 log_10_ copies/10 larvae) (Fig. 1A). Furthermore, we evaluated the impact of FB infection alone on *ifn* and *mx* expression. Compared to the PBS control, FB-infected larvae exhibited only minor changes in the expression of *ifn* and *mx* across the tested time points (6, 24, and 48 hpi, Fig. S3), with fold changes of ~2.59 and ~1.22, respectively, at 48 hpi. This indicated that FB alone could not significantly upregulate the expression of these genes. Therefore, the reduced immune activation in the FB pre-incubated-GII.4 HuNoV group is more likely due to suppressed viral replication. This distinguishes FB from many other antiviral agents that typically enhance host immunity to inhibit viral replication (4). For instance, fucoidan has been reported to inhibit HuNoV replication by enhancing the host innate immune response (38). The injection of fucoidan together with HuNoV upregulated 834 genes in zebrafish larvae (*P* < 0.01), a gene expression pattern similar to that observed with fucoidan alone. Other studies report similar immune-enhancing effects in murine macrophages: neoagarohexaose inhibited MNV replication while boosting IFN-beta production, strengthening antiviral responses (39). Likewise, *L. salivarius* HHuMin-U significantly inhibited MNV replication and enhanced IFN-stimulated gene expression in macrophages (40), while strains such as *L. ruminis* SPM 1308, *L. fermentum* KCTC 3112, *L. rhamnosus* KCTC 18,427P, and *L. reuteri* KCTC 18,428P significantly upregulated *IFN-β* and *IFN-γ* expression in RAW264.7 cells (41). In contrast, the antiviral effect of *L. rhamnosus* YQ001 may offer advantages for therapeutic applications where excessive immune activation is undesirable.

The cellular receptors for HuNoVs remain unidentified. Histo-blood group antigens (HBGAs) are traditionally recognized as attachment receptors or factors for HuNoVs (42). However, some HuNoV genotypes, like GII.1, could infect cells without HBGAs interaction (43). HBGAs-like polysaccharides found in leafy greens, bivalves, and even certain bacteria could aid HuNoV binding and transmission (44–46). Non-HBGAs attachment factors, such as Histone H1 and sialylated glycans, have been shown to block HBGAs binding to HuNoVs and potentially offer therapeutic applications by preventing viral entry (47, 48). Recent studies also highlighted proteinaceous attachment factors in food matrices, such as roHSP70, recombinant oyster tumor necrosis factor, and recombinant oyster intraflagellar transport protein in oyster tissues (49, 50). These proteinaceous attachment factors could bind HuNoV P protein and intact virions, playing critical roles in viral transmission. Various bacteria could bind to HuNoVs, including *L. acidophilus*, *L. bulgaricus*, and *L. reuteri* (51), as well as several common human intestinal bacteria (52). *Escherichia coli* Nissle 1917 and *L. casei* BL23 could bind to HuNoV P protein and inhibit its attachment to HT-29 cells, potentially preventing viral attachment to host receptors (25). However, the specific viral attachment factors on bacterial surfaces remain unidentified. In this context, we found that *L. rhamnosus* YQ001 could directly bind to GII.4 HuNoVs and further identified specific membrane proteins on their surface as potential attachment factors. By binding to viral capsids, these proteins could potentially enhance the antiviral activity of other components present in the FB, providing new insights into antiviral development.

The current study identified three membrane proteins C2JVE6, C2JX39, and C2K0J4 that could act as attachment factors for HuNoV capsid proteins. C2JVE6, an ATP-dependent zinc metalloprotease FtsH, is crucial for bacterial protein quality control and stress response, forming hexameric membrane complexes that degrade both membrane-bound and cytoplasmic proteins (53). C2JX39, classified as DNA topoisomerase VI subunit A, is part of bacterial topoisomerase VI, which plays a critical role in DNA replication and transcription (54). C2K0J4, a penicillin-binding protein, is a key enzyme in bacterial cell wall synthesis and maintenance, facilitating peptide cross-linking for structural integrity (55). The ELISA results confirmed that rC2JVE6, rC2JX39, and rC2K0J4 could strongly bind to both GII.4 and GI.1 P proteins, with efficiencies comparable to known attachment factors such as PGM (42) and roHSP70 (50) (Fig. 8). Similar cross-genogroup binding abilities have been reported for other HuNoV attachment factors such as HBGAs (56) and bile acids (57). HBGAs could bind to both GI and GII HuNoVs, although the binding sites differ between genogroups. GI HuNoVs mainly interact with the Gal moiety of HBGAs through a single subunit of the P protein dimer, while GII HuNoVs primarily bind the Fuc moiety at the interface between two subunits (56). Bile acids have been shown to bind GII.1, GII.10, and GII.19 HuNoV capsids, but not GI.1, GII.3, GII.4, or GII.17. In the bile acid-binding genotypes, two conserved residues formed a hydrophobic surface that complements the hydrophobic core of the bile acid molecule, facilitating stable interaction (57). Given that rC2JVE6, rC2JX39, and rC2K0J4 bound to both GI.1 and GII.4 HuNoV P proteins with comparable affinity, it is plausible that these proteins recognize conserved structural features shared across multiple genotypes, similar to the binding mechanism of bile acids. Their binding ability to diverse HuNoV genotypes highlights their potential as broad-spectrum binding agents, which could be valuable for developing antiviral strategies against genetically diverse HuNoVs.

The P2 subdomain of GII.4 HuNoVs contains two key binding sites for HBGAs, located within a conserved central binding pocket at positions Ser121-Arg123 and in a variable surrounding region at Ser171-Thr173 (58). For roHSP70, binding occurs at His198, Gln41, Asn193, and Thr206 (59). The binding sites between C2JVE6/C2JX39/C2K0J4 and GII.4 HuNoV P2 subdomain are detailed in Table S1. Notably, the Lys505 residue of C2JVE6 interacts with Ser171 of the P2 subdomain, a site known to bind HBGAs. Additionally, the Arg380 residue of C2K0J4 targets Asn193 in the P2 subdomain, mirroring the interaction of Asn334 from roHSP70. ELISA confirmed that rC2JVE6, rC2JX39, and rC2K0J4 exhibited stronger binding to the GII.4 P protein than PGM (Fig. 8). The enhanced binding affinity could arise from the broader range of interaction sites these proteins have with the P2 subdomain, compared to the limited binding sites on HBGAs. The identified proteins C2JVE6, C2JX39, and C2K0J4, considering their high affinity for the HuNoV capsid protein, may serve as key components in a targeted strategy to block viral attachment and entry into host cells, offering a novel avenue for antiviral development.

Although identified membrane proteins (C2JVE6, C2JX39, and C2K0J4) could bind to GII.4 HuNoVs, they did not inhibit viral replication *in vivo* (data not shown). This is consistent with our findings in the current study that *L. rhamnosus* YQ001 cells alone could not inhibit GII.4 HuNoV replication. The replication cycle of HuNoVs is a complex process that includes cell attachment, internalization, uncoating, translation, genomic RNA replication, and virion assembly and release (60). Our findings suggest that although these membrane proteins could physically interact with the virus and potentially interfere with initial host cell attachment, their binding is not sufficient to suppress viral replication *in vivo*. Currently, anti-norovirus drug development mainly targets key enzymes involved in viral replication. For instance, rupintrivir inhibits viral proteases (61), while CM521 and 2′-C-methylcytidine function as RNA-dependent RNA polymerase inhibitors, directly blocking viral genome replication (62, 63). Therefore, we hypothesize that the antiviral effect of the *L. rhamnosus* YQ001 FB may involve components acting beyond the initial attachment stage of the HuNoV life cycle.

The current study provides valuable insights into the antiviral properties of *L. rhamnosus* YQ001 against GII.4 HuNoVs. However, certain limitations persist. First, while the zebrafish model provides a convenient and reproducible system for studying HuNoV replication (6), the translation of these findings to human systems remains a critical next step. Zebrafish larvae possess only an innate immune system, lacking the adaptive immunity found in humans (64). Future research should focus on validating these antiviral mechanisms in mammalian models and examining the potential of *L. rhamnosus* YQ001 to prevent and/or treat HuNoV infections in real-world settings. Second, although we identified antiviral properties of *L. rhamnosus* YQ001, the specific antiviral components in CFS originated from *L. rhamnosus* YQ001 remain to be elucidated. Considering the potential application of *L. rhamnosus* YQ001-derived products—such as FB, CFS, or purified proteins—in functional foods or therapeutic formulations, further investigation is warranted. A key consideration is that *L. rhamnosus* YQ001 belongs to the *L. rhamnosus* species, many strains of which are included in the Qualified Presumption of Safety list by the European Food Safety Authority and are widely used in food and health-related products (65). This provides a promising foundation for its further development. Importantly, our findings imply a potential synergistic effect between the identified membrane proteins and the antiviral substances present in the CFS in inhibiting HuNoV replication. This suggests the feasibility of developing postbiotic-based interventions derived from *L. rhamnosus* YQ001. According to the consensus definition, postbiotics refer to “a preparation of inanimate microorganisms and/or their components that confers a health benefit on the host” (66). Leveraging this concept could facilitate the creation of standardized and highly controllable antiviral formulations, with practical advantages for food and therapeutic applications. In parallel, safety assessments of *L. rhamnosus* YQ001 are underway in our laboratory, and future studies will continue to explore and report on its application potential.

### Conclusion

The current study provides new insights into the antiviral properties of *L. rhamnosus* YQ001 against GII.4 HuNoVs, demonstrating its potential as a probiotic-based antiviral agent. The FB of *L. rhamnosus* YQ001 exhibited robust anti-HuNoV activity, significantly reducing viral replication in zebrafish larvae, with greater efficacy than the CFS. By suppressing viral replication, FB reduced the overall viral load, leading to a decrease in the upregulation of virus-induced immune genes (*ifn* and *mx*). Proteomic and molecular docking analyses identified three membrane proteins, C2JVE6, C2JX39, and C2K0J4, as critical attachment factors that bind to HuNoV capsid proteins, offering a possible mechanism for viral inhibition. This dual effect, combining viral inhibition and viral binding, paves the way for its application in functional foods or therapeutic strategies aimed at reducing the burden of HuNoV infections. Future studies should focus on elucidating the molecular mechanisms involved and validating these findings in mammalian models and clinical settings.

## MATERIALS AND METHODS

### Antiviral activity of *L. rhamnosus* YQ001 against GII.4 HuNoVs

#### Cultivation of *L. rhamnosus* YQ001 and preparation of its derived FB, CFS, and cells

*L. rhamnosus* YQ001 was initially cultured from a single colony in 10.0 mL of MRS medium (Huankai Microbial Co., Ltd., Guangzhou, China) and incubated at 37°C under static conditions for 24 h. The composition of MRS medium (per liter) was as follows: casein peptone, 10.0 g; beef extract, 10.0 g; yeast extract, 4.0 g; ammonium citrate, 2.0 g; sodium acetate, 5.0 g; dipotassium phosphate, 2.0 g; glucose, 20.0 g; magnesium sulfate (MgSO_4_·7H_2_O), 0.2 g; manganese sulfate (MnSO_4_·4H_2_O), 0.05 g; and Tween 80, 1.0 g. A 1:100 dilution of this initial culture was then transferred into 10.0 mL of fresh MRS medium and cultured for an additional 24 h under the same conditions. The concentration of the resulting FB was approximately 1.8 × 10³ CFU/nL, as determined by the spread plate method under anaerobic conditions. The FB of *L. rhamnosus* YQ001 was centrifuged (10,000 × *g*, 10 min) to collect the cells and the CFS. The CFS was filtered through a 0.22 µm filter membrane. The cells were washed and resuspended in an equal volume of PBS (Sangon Biotech Co., Ltd., Shanghai, China) to a final concentration of approximately 1.8 × 10³ CFU/nL, consistent with the initial FB concentration.

#### Zebrafish maintenance and injection of zebrafish larvae with HuNoVs

Wild-type AB adult zebrafish (*D. rerio*) were maintained in the aquatic facilities at 28°C under a 14/10-h light/dark cycle. The embryos and larvae were maintained at 28°C in fish water (48.0 mg/L marine salt and 0.5 mg/L methylene blue).

The injection was performed according to Van Dycke et al. (15), with slight modifications. Three-day-post-fertilization zebrafish larvae were anesthetized in 0.8 mg/mL tricaine (Titan, Shanghai, China). Viral suspension (3 nL, 2.94 × 10^3^ RNA copies) was microinjected into the yolk using a calibrated microinjection system with pulled glass capillaries. For groups receiving bacterial treatments, the viral suspension was pre-incubated with the FB, CFS, or bacterial cell suspension of *L. rhamnosus* YQ001 at a 1:1 ratio for 1 h at room temperature. Both the FB and bacterial cell suspension were adjusted to a final concentration of 1.8 × 10^3^ CFU/nL, resulting in a dose of approximately 5.4 × 10^3^ CFU per larva. Control groups received either PBS, heat-inactivated HuNoVs (95°C, 5 min) or FB (5.4 × 10^3^ CFU per larva).

After injection, the larvae were maintained in fish water at 32°C under a 14/10-h light/dark cycle. Health was monitored daily, with non-viable larvae removed and water refreshed. At 0, 1, and 2 dpi, groups of 10 larvae were collected and stored at −80°C for subsequent analysis.

#### Tissue homogenization, RNA extraction, and RT-qPCR for HuNoV detection

The harvested zebrafish larvae were homogenized using a homogenizer (Eppendorf, Berzdorf, Germany) with three cycles of 30 s at 65 Hz, allowing 60 s rest intervals. RNA was extracted using the FastPure Viral RNA Mini Kit (Vazyme, Nanjing, China) following the manufacturer’s protocol.

Detection of GII.4 HuNoVs was carried out using a One Step qRT-PCR Probe Kit (Vazyme, Nanjing, China) on a qPCR system (eQ9600, Eastwin, Beijing, China). Details of the primers and RT-qPCR reaction system are provided in Tables S2 and S3. The cycling conditions consisted of an initial reverse transcription phase at 50°C for 15 min, followed by denaturation at 95°C for 5 min. Subsequently, 45 amplification cycles were conducted, each consisting of 95°C for 15 s and 60°C for 30 s. The standard curve of GII.4 HuNoV recombinant plasmid is shown in Fig. S7.

#### Evaluation of GII.4 HuNoV capsid recognition of PGM after exposure to *L. rhamnosus* YQ001 by *ISC*-RT-qPCR

The *ISC*-RT-qPCR assay followed an adapted protocol (67). This method was specifically designed to evaluate the impact of different treatments on the integrity of HuNoVs by measuring the ability of viral particles to bind to PGM. First, PCR plate wells (VWR, Brisbane, CA, USA) were coated overnight with 100.0 µL capture unit solution (1.0 mg/mL PGM [Sigma, Saint Louis, MO, USA]) or negative control (1.0% bovine serum albumin [BSA, Vazyme, Nanjing, China]) at 4°C, as described in our previous studies (67, 68). Then, the wells were blocked with 1.0% BSA for 1 h at 37°C, followed by washing with tris-buffered saline with Tween 20 (TBST, Sangon Biotech Co., Ltd.) to remove unbound blocking agent. Concurrently, viral samples were prepared. GII.4 HuNoVs were incubated with their respective treatments (FB, CFS, or bacterial cells) for 1 h at room temperature as described in “Cultivation of *L. rhamnosus* YQ001 and preparation of its derived FB, CFS, and cells,” above. Untreated viral samples were used as controls. Subsequently, the pre-incubated viral samples (100.0 µL) were added to the coated wells and incubated for 1 h at 37°C to allow for virus-PGM binding. The plates were then washed with TBST to remove unbound viral particles. Diethyl pyrocarbonate water (10.0 µL, Sangon Biotech Co., Ltd.) was added to each well, followed by heating at 95°C for 5 min to lyse the captured viruses and release their RNA. The released RNA was then quantified using RT-qPCR as described in “Tissue homogenization, RNA extraction, and RT-qPCR for HuNoV detection,” above.

#### mRNA expression of genes in zebrafish following GII.4 HuNoV infection

RT-qPCR was used to determine the expression levels of the zebrafish genes (i.e., *ifn*, *mx*, and the housekeeping genes *β-actin* [6, 9]). The stability of the *β-actin* reference gene was validated across all experimental conditions, confirming its suitability as a stable reference gene for normalization. The RNA of zebrafish larvae was extracted using the RNA Isolation Kit (Vazyme, Nanjing, China) following the manufacturer’s protocol. Primers and reaction system of RT-qPCR are previously described (69) and are shown in Tables S4 and S5. Cycling conditions were: reverse transcription at 50°C for 15 min, polymerase activation at 95°C for 3 s, followed by 40 cycles of denaturation at 95°C for 10 s, annealing and extension at 60°C for 30 s. The fold induction of gene expression was determined by the 2^ΔΔCT^ method with housekeeping genes for normalization.

#### Bacterial enumeration

The bacterial enumeration was performed according to Toh et al. (9). *L. rhamnosus* YQ001 was cultured as described in “Cultivation of *L. rhamnosus* YQ001 and preparation of its derived FB, CFS, and cells,” above, and was microinjected into the yolk as described in “Zebrafish maintenance and injection of zebrafish larvae with HuNoVs,” above, delivering approximately 5.4 × 10^3^ CFU per larva upon injection. To assess the potential *in vivo* proliferation of *L. rhamnosus* YQ001, zebrafish larvae were collected at 6, 24, and 48 hpi, in groups of 10 per replicate. Immediately after collection, the larvae were submerged in ice water for 10 min for euthanasia, then homogenized as described in “Tissue homogenization, RNA extraction, and RT-qPCR for HuNoV detection,” above. The resulting homogenates were serially diluted in PBS and plated on MRS agar containing vancomycin (50.0 µg/mL). This selective medium allows for the growth of the injected *L. rhamnosus* YQ001, which is vancomycin-resistant, while suppressing the growth of other environmental contaminants (70, 71).

### Evaluation of *L. rhamnosus* YQ001 binding to GII.4 HuNoV P protein

#### Purification of GII.4 HuNoV P protein and roHSP70

*E. coli* BL21 (Thermo Fisher, Shanghai, China) was used as the competent cell for recombinant plasmid transformation and for the expression of GII.4 HuNoV P protein and roHSP70. pET-28a-P (GII.4) and pET-28a-roHSP70 were stored in our laboratory (50, 72). The protein purification was conducted using Ni-nitrilotriacetic acid beads 6FF (Smart-Lifesciences, Changzhou, China) following our previous study (50).

#### Binding of GII.4 HuNoV P protein to *L. rhamnosus* YQ001

The binding interaction between the GII.4 HuNoV P protein and *L. rhamnosus* YQ001 was analyzed by Western blot (51). *L. rhamnosus* YQ001 was cultured in MRS medium at 37°C under static conditions until the OD_600_ reached 0.6. The cells were collected, washed, and resuspended in PBS to an OD_600_ of 1.0. Then, 50.0 µg of P protein was incubated with 1.0 mL bacterial suspension at 37°C for 1 h. Following incubation, the mixture was centrifuged, and the bacterial cells were washed thoroughly with PBS and dissolved in SDS-PAGE loading buffer (Servicebio, Wuhan, China). After boiling for 10 min, samples were loaded to 4–14% SDS-PAGE gel. The proteins on gels were transferred to the polyvinylidene fluoride (PVDF) membranes (Beyotime, Shanghai, China) using a wet tank transfer system (90 V, 140 min, Bio-Rad, Hercules, CA). The PVDF membranes were blocked with 5% skim milk for 1 h at room temperature, then incubated overnight at 4°C with primary antibodies (mouse monoclonal anti-HuNoV GII.4, 1:2,000 dilution, stored in our lab [72]). After washing with TBST, the membranes were incubated with secondary antibodies (polyclonal goat anti-mouse-HRP, 1:2,000 dilution; Sangon Biotech Co., Ltd.) for 1 h at room temperature, followed by washing with TBST. Immunoreactive bands were visualized using the QuickChemi 5200 system (Monad Biotech Co., Ltd., Shanghai, China) with the BeyoECL Star Kit (Beyotime).

#### Binding of GII.4 HuNoV P protein to *L. rhamnosus* YQ001 membrane proteins

After 24 h of static culture, *L. rhamnosus* YQ001 cells were centrifuged (10,000 × *g*, 10 min) to isolate bacterial membrane proteins following the manufacturer’s instructions (Signalway antibody LLC, Greenbelt, MD). Bacterial membrane proteins were dissolved in SDS-PAGE loading buffer (Servicebio) and boiled for 10 min. Samples were loaded to 4–14% SDS-PAGE gel. To elucidate the binding ability of HuNoVs to bacterial membrane proteins, a virus overlay protocol was adapted from Almand et al. (26). The proteins on gels were transferred to PVDF membranes and blocked as described in “Binding of GII.4 HuNoV P protein to *L. rhamnosus* YQ001,” above. The PVDF membranes were then incubated with GII.4 HuNoV P protein (50.0 µg/mL) at 4°C overnight. After washing with TBST, the P protein was detected using the same antibody and visualized as described in “Binding of GII.4 HuNoV P protein to *L. rhamnosus* YQ001,” above.

### Identification of proteinaceous attachment factors

#### PVDF membrane preparation for mass spectrometry

Excised PVDF membrane strips were incubated with 10.0 mM dithiothreitol at 37°C for 1 h, followed by treatment with 25.0 mM iodoacetamide in the dark at room temperature for 20 min. After washing with 50.0 mM ammonium bicarbonate, the membranes underwent trypsin digestion (1.0 ng/µL, Promega, Madison, WI) at 37°C. The resulting peptides were collected, dried, desalted using Empore SDB-RPS StageTips, and dissolved in 10.0 µL of 0.1% formic acid for analysis in “Mass spectrometry for protein analysis,” below.

#### Mass spectrometry for protein analysis

Proteomic analysis was performed using an Orbitrap Exploris 480 mass spectrometer (Thermo Fisher Scientific, Waltham, MA) in full-scan and data-dependent MS/MS (ddMS²) modes. Full scans were acquired in positive ion mode (*m/z* 350–1,500) at a resolution of 60,000, with an AGC target of 300% and a maximum injection time of 20 ms. Data-dependent MS/MS scans were performed at a resolution of 15,000, using a 1 *m/z* isolation window, a 30% collision energy for higher-energy collisional dissociation, an AGC target of 100%, and a maximum injection time of 20 ms.

Peptide separation was achieved on a C18 reverse-phase column (Thermo Fisher Scientific) at a flow rate of 300 nL/min. The mobile phases consisted of Buffer A (0.1% formic acid in water) and Buffer B (80% acetonitrile with 0.1% formic acid). The gradient program was: 2–8% Buffer B in 2 min, 8–30% in 48 min, 30–50% in 3 min, 50–90% in 1 min, and held at 90% for 6 min.

#### Molecular docking of GII.4 HuNoV P protein and attachment factors

The structure of the GII.4 HuNoV P protein was obtained from the PDB (https://www.rcsb.org/). The PDB ID of the P protein is 4OOX (73). The sequence and structure of membrane protein were retrieved from the UniProt database (https://www.uniprot.org/).

Molecular docking between P protein and candidate proteins was performed using GRAMM-X (74) and AlphaFold 3 (75). The top-scoring docking results were analyzed using PDBePISA (http://www.ebi.ac.uk/pdbe/prot_int/pistart.html) to investigate the interaction energies and forces at the interface. The results were visualized using Pymol for clear representation.

### Evaluation of the viral binding ability of preferred attachment factors

#### Prokaryotic expression and purification of preferred attachment factors

The nucleic acid sequences of selected candidate proteins (UniProt accession numbers: C2JVE6, C2JX39, and C2K0J4) were analyzed by online tools SignalP-4.0 (https://services.healthtech.dtu.dk/services/SignalP-4.1/) and DeepTMHMM (https://services.healthtech.dtu.dk/services/TMHMM-2.0/), with transmembrane and signal peptide regions excised. The remaining extracellular region amino acid sequences were used for subsequent analyses. The DNA of *L. rhamnosus* YQ001 was extracted and used as a template to obtain the gene fragments of C2JVE6, C2JX39, and C2K0J4. Detailed information about the designed primers and reaction system of PCR is shown in Table S6 and S7. The thermo-cycling parameters were as follows: 95°C for 3 min, followed by 35 cycles of 95°C for 30 s, 60°C for 30 s, 72°C for 90 s, and a final step at 72°C for 5 min. The amplified PCR products were separately inserted into the pET-22b(+) plasmid. The recombinant plasmids were transformed into *E. coli* BL21 for protein expression and purification as described in “Purification of GII.4 HuNoV P protein and roHSP70,” above.

#### Evaluation of the binding ability of rC2JVE6/rC2JX39/rC2K0J4 to P protein by ELISA

The purified proteins (rC2JVE6, rC2JX39, and rC2K0J4) were diluted to 280.0 pmol/mL in carbonate-bicarbonate buffer and added to ELISA plate wells for overnight incubation at 4°C. Controls included 1.0% BSA (negative), 1.0 mg/mL PGM (positive), and 280.0 pmol/mL roHSP70 (positive). After washing with PBS, wells were blocked with 1.0% BSA for 1 h at 37°C and washed with TBST. P protein (GI.1 or GII.4, 10.0 µg/mL, 100.0 µL) was added and incubated for 1 h at 37°C. After TBST washes, mouse monoclonal anti-HuNoV GI.1 or GII.4 (1:3,000, stored in our lab [72]) and goat anti-mouse-HRP antibodies (1:2,000; Sangon Biotech Co., Ltd.) were sequentially applied, each for 1 h at 37°C, with washes in between. 3,3′,5,5′-tetramethylbenzidine (TMB, Frdbio, Wuhan, China) was added and incubated for 10 min in the dark, followed by 2.0 M H_2_SO_4_ to stop the reaction. Absorbance was measured at 450 nm using a Multiskan FC microplate photometer (Thermo Fisher Scientific). The samples were considered positive when the sample S/N ratio was equal to or greater than 2.0.

#### Evaluation of the binding ability of rC2JVE6/rC2JX39/rC2K0J4 to GII.4 HuNoV by *ISC*-RT-qPCR

Wells of PCR reaction plates (VWR) were coated with 100.0 µL of capture unit solution (1.0 mg/mL PGM; 280.0 pmol/mL roHSP70, rC2JVE6, rC2JX 39, and rC2K0J4; 1.0% BSA). Then, the plates were incubated overnight at 4°C. The subsequent steps were performed as described in “Evaluation of GII.4 HuNoV capsid recognition of PGM after exposure to *L. rhamnosus* YQ001 by *ISC*-RT-qPCR,” above.

### Statistics

All experiments were performed in triplicate to ensure reproducibility. Data were analyzed by SPSS 26.0, and statistically significant differences were determined with *P* values less than 0.05.

## SUPPLEMENTAL MATERIAL

Supplemental material aem.01046-25-s0001.docx Fig. S1 to S7; Tables S1 to S7.

ASM does not own the copyrights to Supplemental Material that may be linked to, or accessed through, an article. The authors have granted ASM a non-exclusive, world-wide license to publish the Supplemental Material files. Please contact the corresponding author directly for reuse.
