# Supplementary material for: Lactobacillus rhamnosus YQ001 binds with GII.4 human noroviruses and inhibits viral replication in zebrafish larvae
Source: Appl Environ Microbiol. 2025 Oct 16;91(11):e01046-25. doi: 10.1128/aem.01046-25 (PMC12628836; doi:10.1128/aem.01046-25)
Supplement: Supplemental material — Fig. S1 to S7; Tables S1 to S7. [file aem.01046-25-s0001.docx]

***Lactobacillus rhamnosus* YQ001 Binds with GII.4 Human Noroviruses and Inhibits the Viral Replication in Zebrafish Larvae**

Yaqi Yang,^a, *^ Ran An,^a, *^ Xiangjun Zhan,^a^ Yunce Liu,^a^ Mengge Sun,^a^ Shang Chen,^a^ Chenang Lyu,^a^ Yutong Yang,^b,c^ Qinghua Zhang,^b,c^ Lin Yao,^d^ Dapeng Wang^a, #^

^a^ Department of Food Science and Technology, School of Agriculture and Biology, Shanghai Jiao Tong University, Shanghai 200240, China.

^b^ The Key Laboratory of Exploration and Utilization of Aquatic Genetic Resources, Ministry of Education, Shanghai Ocean University, Shanghai 201306, China

^c^ The China National Pathogen Collection Center for Aquatic Animals, Shanghai Ocean University, Shanghai 201306, China

^d^ Yellow Sea Fisheries Research Institute, Chinese Academy of Fishery Sciences, Qingdao 266071, China

Running Head: *L. rhamnosus* YQ001 Binds and Inhibits GII.4 Norovirus

^#^ Corresponding author: Department of Food Science and Technology, School of Agriculture and Biology, Shanghai Jiao Tong University, Shanghai 200240, China. Telephone number: +86 15900710323; E-mail: [dapengwang@sjtu.edu.cn](mailto:dapengwang@sjtu.edu.cn).

^*^ Yaqi Yang and Ran An contributed equally to this work. Author order was determined based on the division of work.

Declarations of interest: All authors disclose no conflicts of interests.

Table S1 The predicted interface forces between attachment factors and GII.4 P protein

| Amino acid residues | | | Bond Length (Å) | Interface forces |
| --- | --- | --- | --- | --- |
| Attachment factors | | GII.4 P protein |  |  |
| C2JVE6 | 25 Asp | 240 Tyr | 3.0 | Hydrogen bonds |
|  | 30 Lys | 94 Glu | 3.4 |  |
|  | 219 Asn | 91 Pro | 2.7 |  |
|  | 245 Asn | 80 Asn | 2.2 |  |
|  | 249 Glu |  | 2.5 |  |
|  | 273 Arg | 78 Thr | 2.9 |  |
|  | 259 Lys | 190 Asn | 2.1 |  |
|  | 505 Lys | 171 Ser | 3.6 |  |
|  | 507 Lys | 132 Gly | 2.6 |  |
|  | 508 Gln | 176 Asn | 2.5 |  |
|  |  | 177 Glu | 3.4 |  |
|  | 504 Asp | 107 Lys | 2.4 | An ionic bond |
| C2JX39 | 51 Gln | 69 Thr | 2.0 | Hydrogen bonds |
|  | 58 Lys | 67 Asp | 2.8 |  |
|  | 61 Arg |  | 2.7 |  |
|  | 60 Phe | 88 Asn | 3.4 |  |
|  | 65 Lys | 80 Asn | 2.1 |  |
|  |  | 81 Leu | 2.4 |  |
|  | 69 Asn | 142 Ser | 2.7 |  |
|  | 57 Asp | 156 His | 3.4 | Ionic bonds |
|  | 58 Lys | 67 Asp | 2.8 |  |
|  | 61 Arg |  | 3.7 |  |
|  |  |  | 3.2 |  |
|  |  |  | 2.7 |  |
| C2K0J4 | 60 Arg | 119 Asp | 3.5 | Ionic bonds |
|  | 187 Lys | 73 Gly | 3.0 |  |
|  |  | 150 Glu | 2.8 |  |
|  | 189 Ser | 150 Glu | 2.1 |  |
|  | 190 Ser |  | 3.3 |  |
|  | 193 Thr | 151 Asn | 2.5 |  |
|  | 199 Trp | 154 Glu | 3.2 |  |
|  | 242 Tyr | 80 Asn | 2.8 |  |
|  | 248 Ala | 66 Gly | 3.3 |  |
|  |  | 157 Gln | 3.3 |  |
|  |  | 158 Asn | 3.0 |  |
|  | 251 Leu | 158 Asn | 3.4 |  |
|  | 253 Lys | 83 Ser | 2.9 |  |
|  |  | 87 Asn | 2.7 |  |
|  | 257 Leu | 117 Lys | 2.1 |  |
|  | 265 Lys | 67 Asp | 2.7 |  |
|  | 270 Gln | 89 Tyr | 3.1 |  |
|  | 273 Asp | 88 Asn | 2.6 |  |
|  | 359 Gln | 74 Ser | 2.9 |  |
|  | 362 Try | 135 Pro | 3.4 |  |
|  | 369 Thr | 190 Asn | 2.8 |  |
|  | 380 Arg | 193 Asn | 3.1 |  |
|  | 477 Thr | 91 Pro | 3.1 |  |
|  |  | 92 Thr | 2.6 |  |
|  |  | 93 Glu | 3.6 |  |
|  | 187 Lys | 150 Glu | 2.8 | Ionic bonds |
|  |  |  | 3.8 |  |
|  | 265 Lys | 67 Asp | 2.7 |  |
|  |  |  | 3.3 |  |

* Transmembrane and signal peptide regions of C2JVE6, C2JX39, and C2K0J4 were excised.

Table S2 Primers and probes sequence used for GII.4 HuNoVs detection

| Viruses | Name | Sequence (5’-3’) | Reference |
| --- | --- | --- | --- |
| GII.4 HuNoVs | QNIF2d | 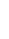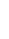ATGTTCAGRTGGATGAGRTTCTCWGA | (1) |
|  | COG2R | TCGACGCCATCTTCATTCACA |  |
|  | QNIFS | FAM- AGCACGTGGGAGGGCGATCG -TAMRA |  |

Table S3 Reaction system of RT-qPCR for GII.4 HuNoVs detection

| Component | Volumes |
| --- | --- |
| 2×One Step Q Probe Mix | 10.0 μL |
| One Step Q probe enzyme Mix | 1.0 μL |
| Primer forward (10.0 μM) | 0.8 μL |
| Primer reverse (10.0 μM) | 0.8 μL |
| Probe (10.0 μM) | 0.4 μL |
| RNA | 2.0 μL |
| RNase-free ddH_2_O | To 20.0 μL |

Table S4 Probes sequence used for mRNA expression

| *Gene* | Accession no. | Primer sequences | Product size (bp) | Reference |
| --- | --- | --- | --- | --- |
| Zebrafish *β-actin* | AF025305 | 5’-ATGGATGAGGAAATCGCTG-3’  5’-ATGCCAACCATCACTCCCTG-3’ | 130 | (2) |
| Zebrafish *mx* | AF533769 | 5’-ATAGGAGACCAAAGCTCGGGAAAG-3’  5’-ATTCTCCCATGCCACCTATCTTGG-3’ | 145 |  |
| Zebrafish *ifn* | AY135716 | 5’-GAATGGCTTGGCCGATACAGGATA-3’  5’-TCCTCCACCTTTGACTTGTCCATC-3’ | 136 |  |

Table S5 Reaction system of RT-qPCR for mRNA expression

| Component | Volumes |
| --- | --- |
| 2×One step SYBR green mix | 10.0 μL |
| One step SYBR green enzyme mix | 1.0 μL |
| Primer forward (10.0 μM) | 0.4 μL |
| Primer reverse (10.0 μM) | 0.4 μL |
| RNA | 20.0 ng |
| RNase-free ddH_2_O | To 20.0 μL |

Table S6 Amplification primers and insertion restriction endonuclease sites of C2JVE6/C2JX39/C2K0J4

| Gene name | Primer name | Restriction enzyme cutting site | Sequence (5’-3’) | Fragment length |
| --- | --- | --- | --- | --- |
| C2JVE6 | C2JVE6-F | *Nco* І | CCCATGGCGATGAATCAGGCAGGTCAAGG | 1686 bp |
|  | C2JVE6-R | *Xho* І | CCTCGAGGGCTTGCGGATTCTGATGAT |  |
| C2K0J4 | C2K0J4-F | *Sac* І | CGAGCTCGCAAGGCAAGGCTGAAG | 1902 bp |
|  | C2K0J4-R | *Xho* І | CCTCGAGGTACAAACTAGCCACAAAAGG |  |
| C2JX39 | C2JX39-F | *Sac* І | CGAGCTCGATGGCCAATCAGATTCAAGAG | 2442 bp |
|  | C2JX39-R | *Xho* І | CCTCGAGCGGTTCAACCGGTGTTTC |  |

Table S7 Reaction system of PCR

| Component | Volumes |
| --- | --- |
| 2×Phanta Max Master Mix | 25.0 μL |
| Primer forward (10.0 μM) | 2.0 μL |
| Primer reverse (10.0 μM) | 2.0 μL |
| Template | 2.0 μL |
| ddH_2_O | To 50.0 μL |

**
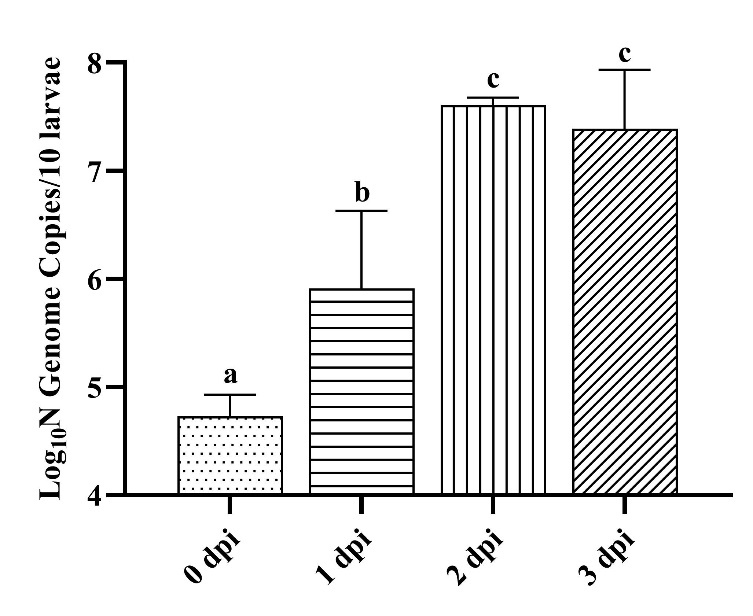
**

**Fig. S1.** **Replication of GII.4 HuNoVs in zebrafish larvae.** Zebrafish larvae were injected with GII.4 HuNoVs. Groups of 10 zebrafish were collected at different time points. Bars represent viral RNA levels, quantified by RT-qPCR (3 independent experiments). Mean values ± standard deviation are presented. Different lowercase letters above the bars indicate statistical significance between groups, as determined by One-way ANOVA test (*p*<0.05). dpi: days post-injection.

**
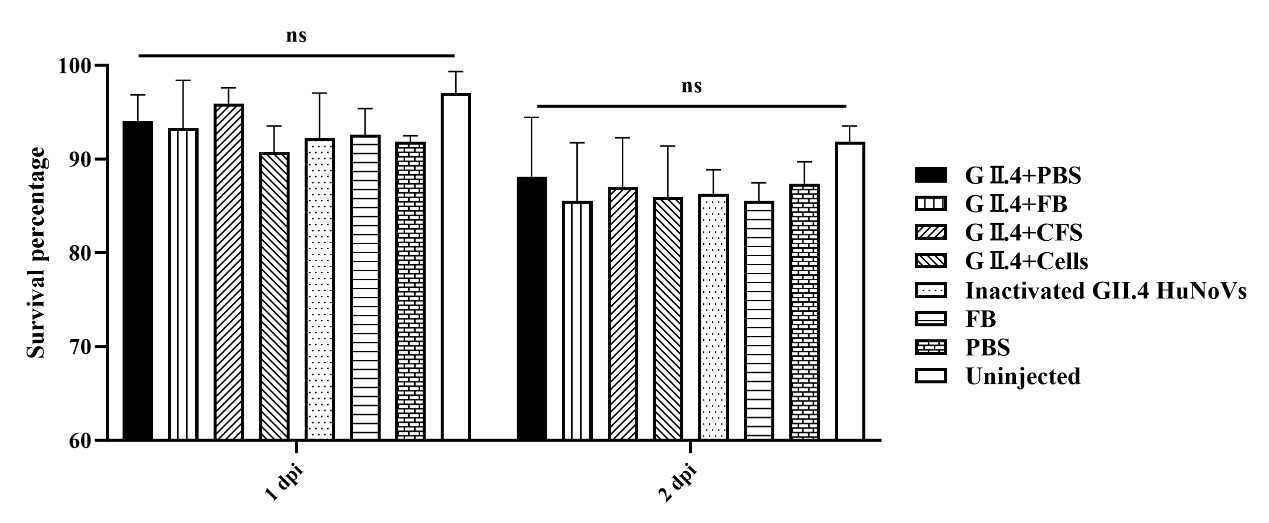
**

**Fig. S2.** **Survival** **percentage of zebrafish larvae after microinjection.** Each bar represents the mean ± standard deviation of three independent experiments (90 larvae per group). No significant differences were observed among the groups (ns, *p*>0.05). FB: fermented broth; CFS: cell-free supernatant; Uninjected: zebrafish larvae without injection; dpi: days post-injection.


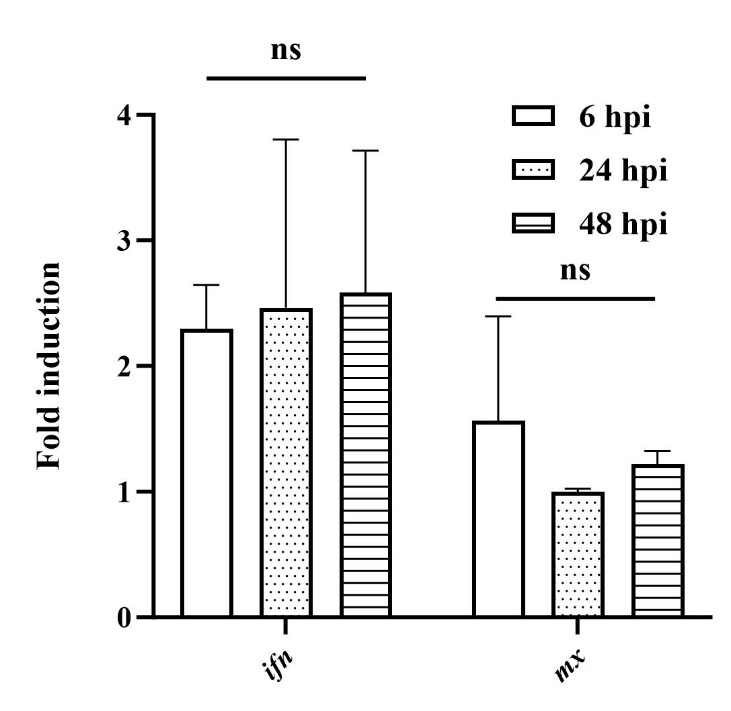


**Fig. S3. Impact of *Lactobacillus rhamnosus* YQ001 fermented broth on *ifn* and *mx* expression.** Gene expression levels were measured by RT-qPCR and normalized to housekeeping genes (3 independent experiments). Bars indicate the relative fold-change in zebrafish larvae injected with *L.* rhamnosus YQ001 fermented broth compared to PBS-injected controls. Mean values ± standard deviation are presented. No significant differences were observed among the groups (ns, *p*>0.05). hpi: hours post-injection.

**Fig. S4. The interface forces between C2JVE6 and GII.4 P protein.** Transmembrane and signal peptide regions of C2JVE6 were excised. Hydrogen bonds are represented by the dashed yellow line and ionic bonds by the dashed green line. Panel A shows that Asp25 and Lys30 of C2JVE6 interact with Tyr240 and Glu94 of the GII.4 P protein to form a hydrogen bond, respectively. Panel B shows that Asn219 of C2JVE6 interacts with Pro91 of the GII.4 P protein to form a hydrogen bond. Panel C shows that Asn245 and Glu249 of C2JVE6 interact with Asn80 of the GII.4 P protein to form two hydrogen bonds. Arg273 of C2JVE6 interacts with Thr78 of the GII.4 P protein to form a hydrogen bond. Panel D shows that Lys259 of C2JVE6 interacts with Asn190 of the GII.4 P protein to form a hydrogen bond. Panel E and F show that Lys505 and Lys507 of C2JVE6 interact with Ser171 and Gly132 of the GII.4 P protein to form a hydrogen bond, respectively. Panel G shows that Gln508 of C2JVE6 interacts with Asn176 and Glu177 of the GII.4 P protein to form a hydrogen bond, respectively. Panel H shows that Asp504 of C2JVE6 interacts with Lys107 of the GII.4 P protein to form an ionic bond.


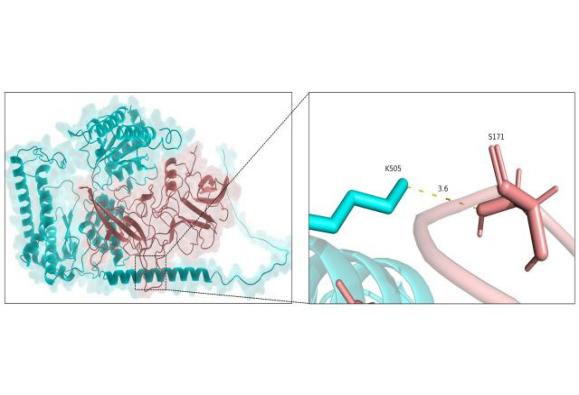

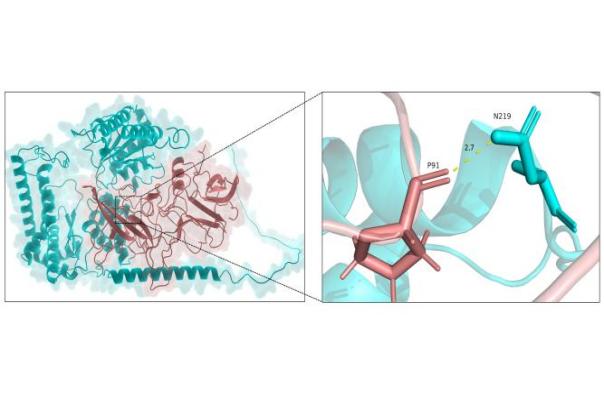

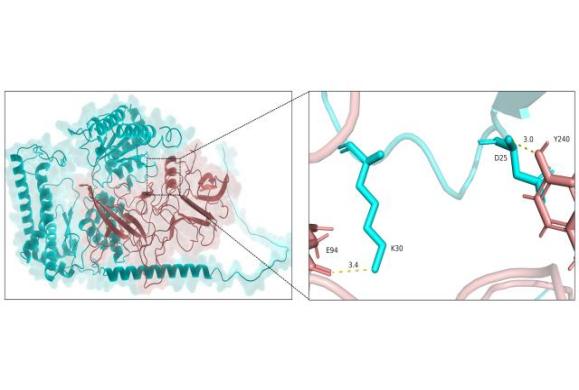


**A**

**B**


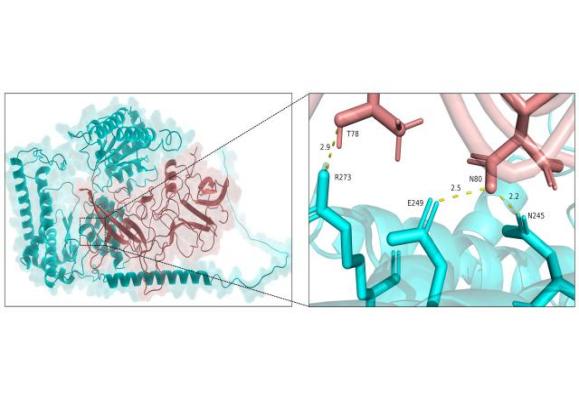


**C**


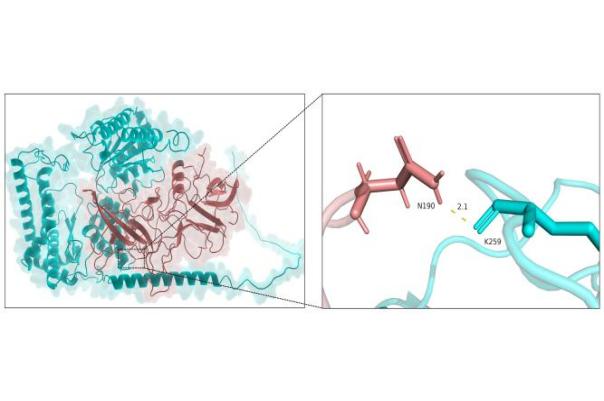


**D**


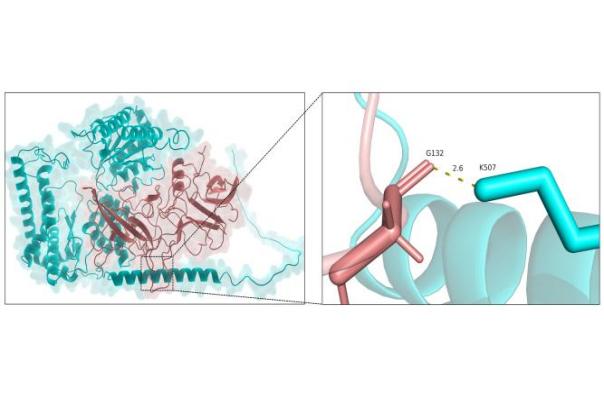


**E**

**F**


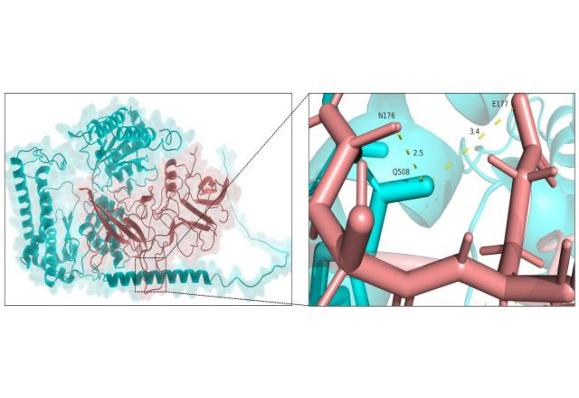


**G**


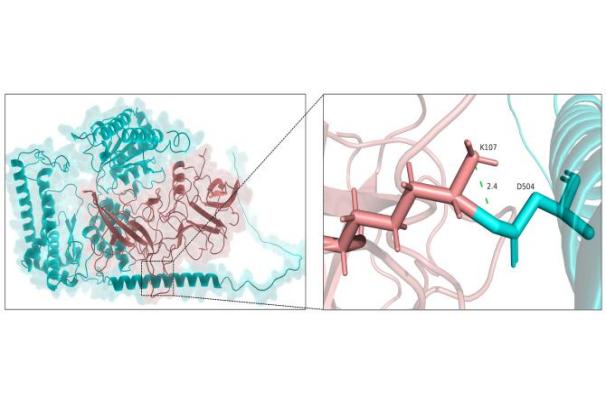


**H**

**B**

**D**

**E**

**F**

**G**

**Fig. S5. The interface forces between C2JX39 and GII.4 P protein.** Transmembrane and signal peptide regions of C2JX39 were excised. Hydrogen bonds are represented by the dashed yellow line and ionic bonds by the dashed green line. Panel A shows that Gln51 of C2JX39 interacts with Thr69 of the GII.4 P protein to form a hydrogen bond. Panel B shows that Lys58 and Arg61 of C2JX39 interact with Asp67 of the GII.4 P protein to form two hydrogen bonds. Panel C shows that Phe60 of C2JX39 interacts with Asn88 of the GII.4 P protein to form a hydrogen bond. Panel D shows that Lys65 of C2JX39 interacts with Asn80 and Leu81 of the GII.4 P protein to form a hydrogen bond, respectively. Panel E shows that Asn69 of C2JX39 interacts with Ser142 of the GII.4 P protein to form a hydrogen bond. Panel F shows that Asp57 of C2JX39 interacts with His156 of the GII.4 P protein to form an ionic bond. Panel G shows that Lys58 of C2JX39 interacts with Asp67 of the GII.4 P protein to form an ionic bond. Arg61 of C2JX39 interacts with Asp67 of the GII.4 P protein to form three ionic bonds.


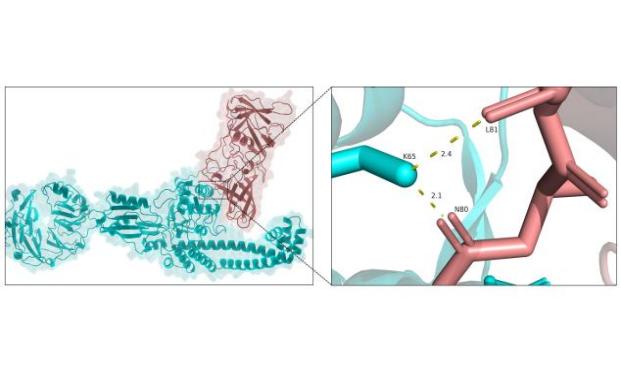

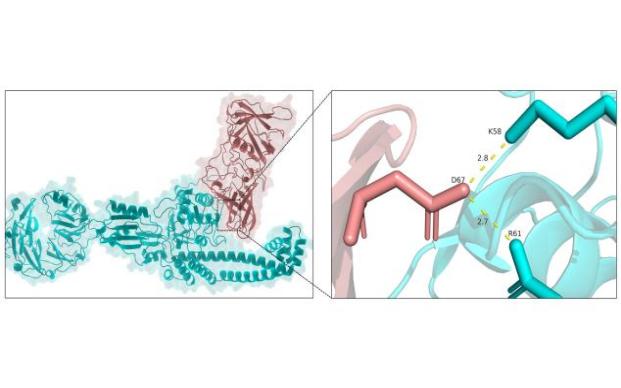

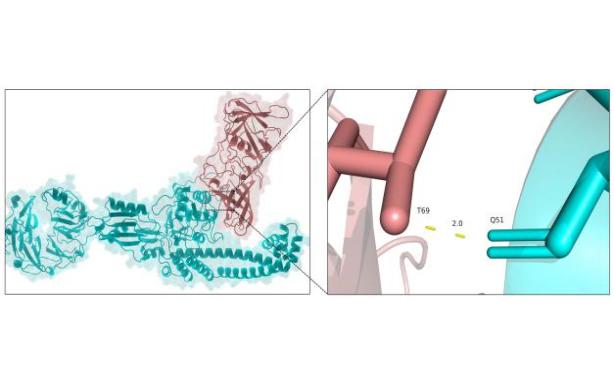


**A**

**B**


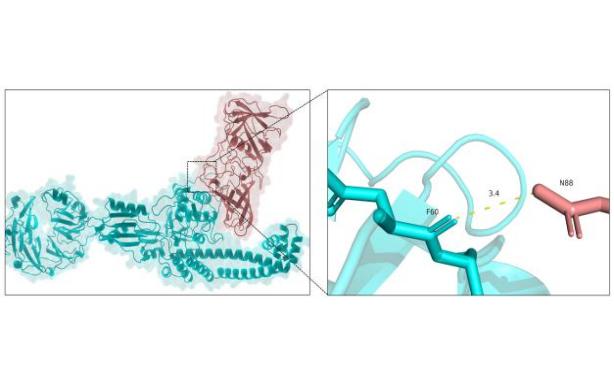


**C**

**D**


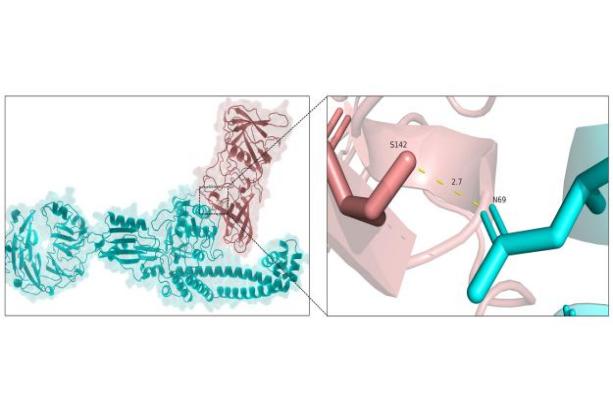


**E**


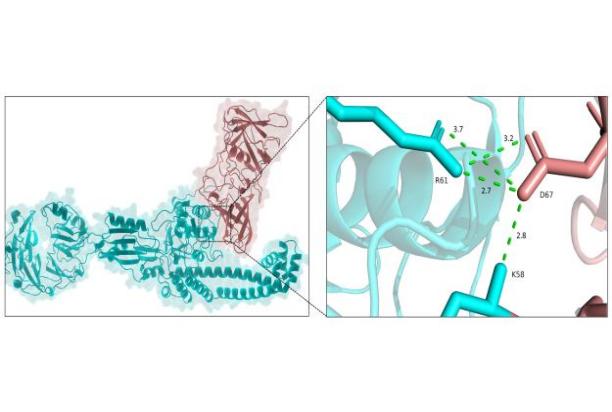


**G**


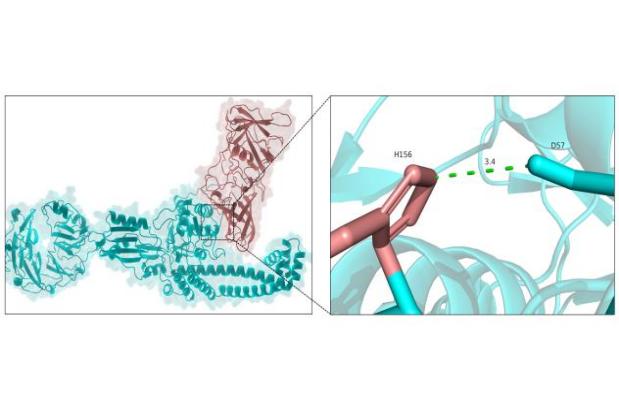


**F**

**Fig. S6. The interface forces between C2K0J4 and GII.4 P protein.** Transmembrane and signal peptide regions of C2K0J4 were excised. Hydrogen bonds are represented by the dashed yellow line and ionic bonds by the dashed green line. Panel A shows that Arg60 and Trp199 of C2K0J4 interact with Asp119 and Glu154 of the GII.4 P protein to form a hydrogen bond, respectively. Panel B shows that Lys187 of C2K0J4 interacts with Gly73 and Glu150 of the GII.4 P protein to form a hydrogen bond, respectively. Ser189 and Ser190 of C2K0J4 interact with Glu150 of the GII.4 P protein to form a hydrogen bond, respectively. Thr193 and Gln359 of C2K0J4 interact with Asn151 and Ser74 of the GII.4 P protein to form a hydrogen bond, respectively. Panel C shows that Tyr242 and Lys265 of C2K0J4 interact with Asn80 and Asp67 of the GII.4 P protein to form a hydrogen bond, respectively. Panel D shows that Ala248 of C2K0J4 interacts with Gly66, Gln157 and Asn158 of the GII.4 P protein to form three hydrogen bonds. Leu251 of C2K0J4 interacts with Asn158 of the GII.4 P protein to form a hydrogen bond. Panel E shows that Lys253 of C2K0J4 interacts with Ser83 and Asn87 of the GII.4 P protein to form a hydrogen bond, respectively. Gln270 and Asp273 of C2K0J4 interact with Tyr89 and Asn88 of the GII.4 P protein to form a hydrogen bond, respectively. Panel F shows that Leu257 of C2K0J4 interacts with Lys117 of the GII.4 P protein to form a hydrogen bond. Panel G shows that Try362 of C2K0J4 interacts with Pro135 of the GII.4 P protein to form a hydrogen bond. Panel H shows that Thr369 and Arg380 of C2K0J4 interact with Asn190 and Asn193 of the GII.4 P protein to form a hydrogen bond, respectively. Panel I shows that Thr477 of C2K0J4 interacts with Pro91, Thr92 and Glu93 of the GII.4 P protein to form three hydrogen bonds. Panel J shows that Lys187 of C2K0J4 interacts with Glu150 of the GII.4 P protein to form two ionic bonds. Panel K shows that Lys265 of C2K0J4 interacts with Asp67 of the GII.4 P protein to form two ionic bonds.


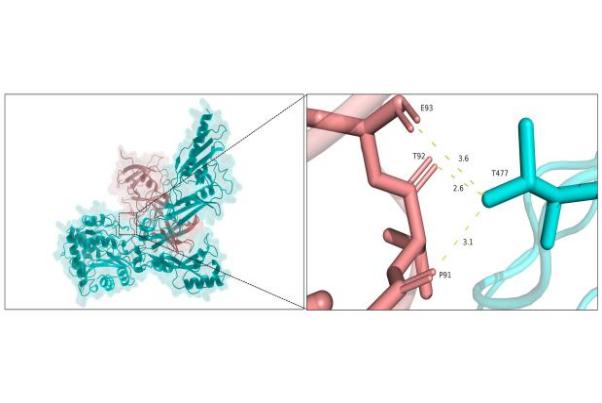

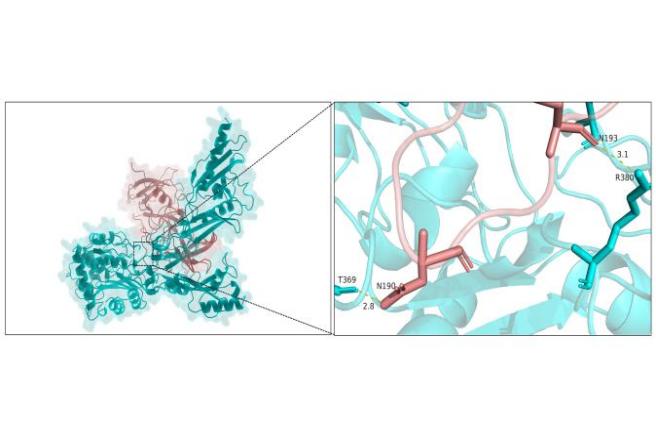

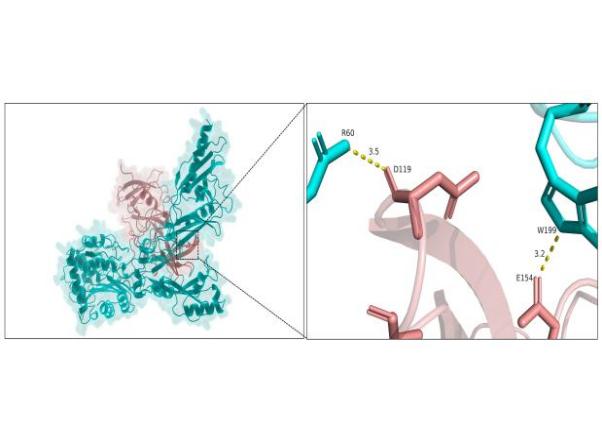


**A**


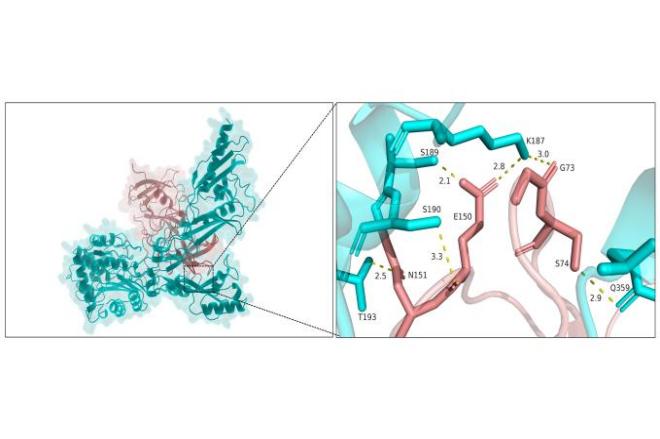


**B**


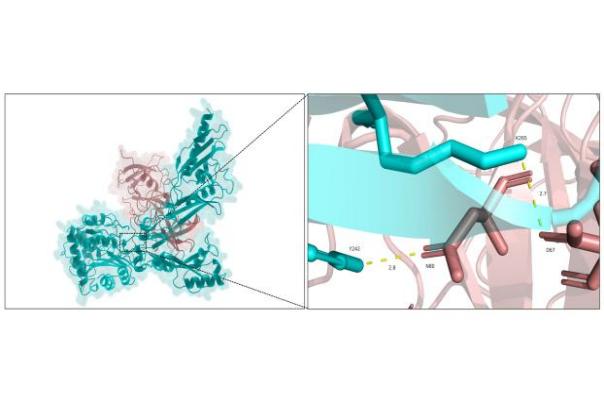


**C**


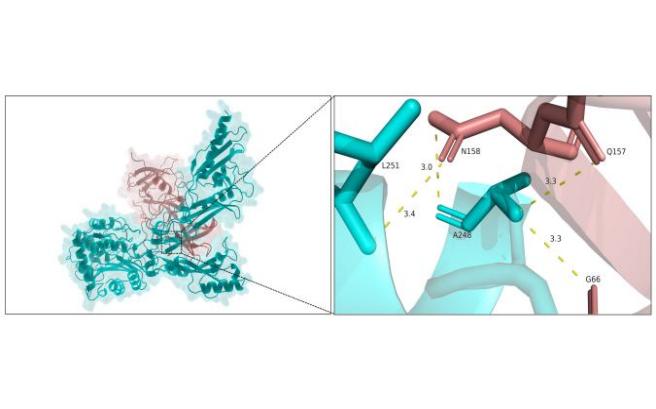


**D**


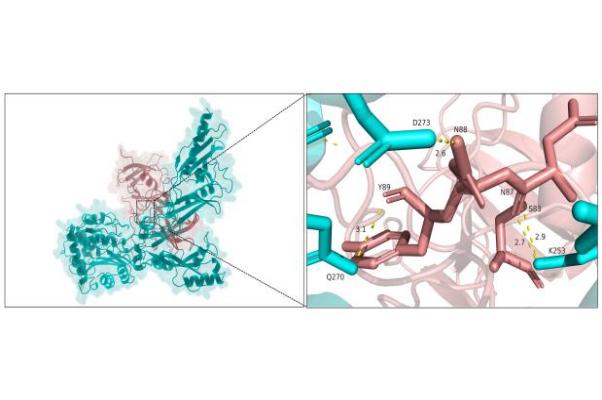


**E**


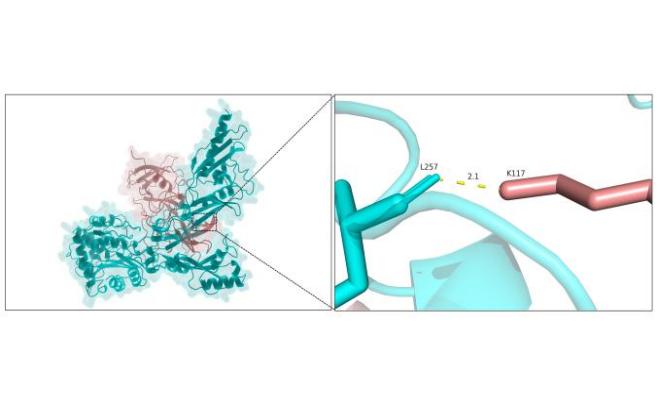


**F**


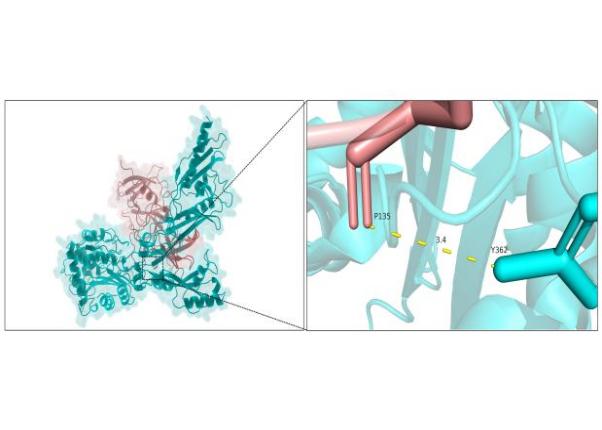


**G**

**H**

**I**


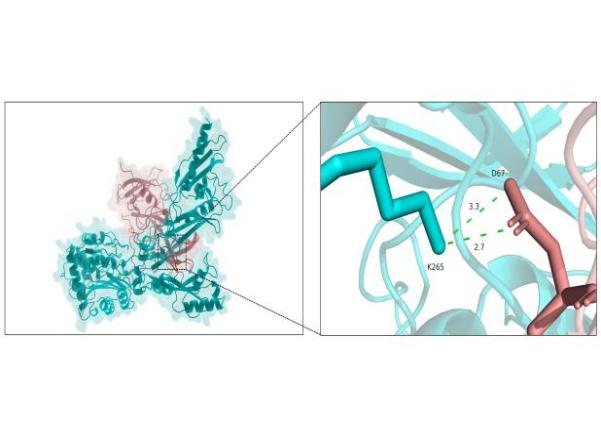

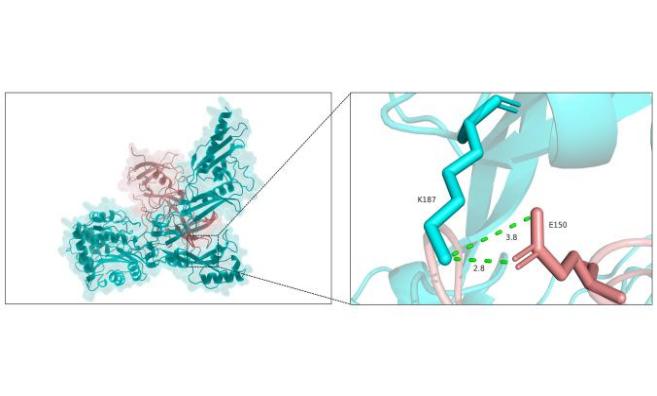


**J**

**K**

**
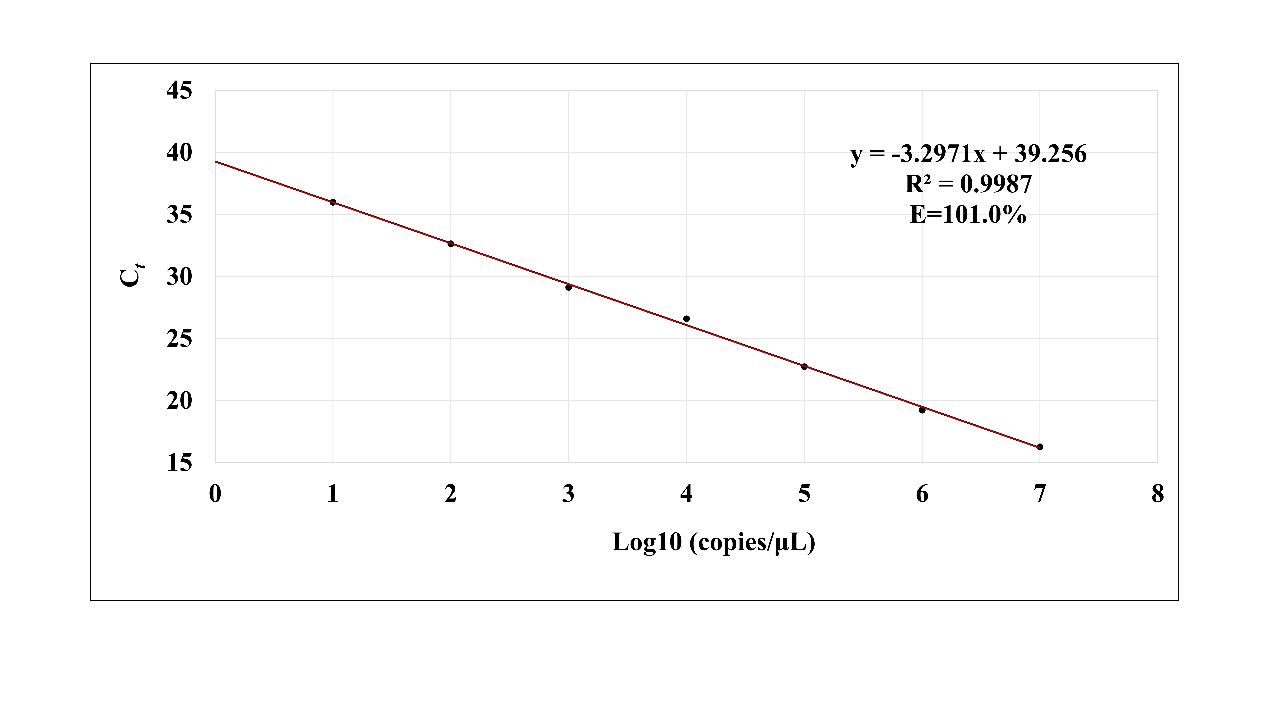
Fig. S7. Standard curve of the recombinant plasmid of GII.4 HuNoVs**

**The amino acid sequence of GII.4 P protein**

GSKPFTVPILTVEEMTNSRFPIPLEKLFTGPSGAFVVQPQNGRCTTDGVLLGTTQLSPVNICTFRGDVTHIAGSRNYTMNLASLNWNNYDPTEEIPAPLGTPDFVGKIQGLLTQTTKGDGSTRGHKATVYTGSAPFTPKLGSVQFSTDTENDFETHQNTKFTPVGVIQDGSTTHRNEPQQWVLPSYSGRNVHNVHLAPAVAPTFPGEQLLFFRSTMPGCSGYPNMDLDCLLPQEWVQHFYQEAAPAQSDVALLRFVNPDTGRVLFECKLHKSGYVTVAHTGQHDLVIPPNGYFRFDSWVNQFYTLAPM

**The amino acid sequence of C2JVE6**

MNQAGQGGGSGRVMSFGKSRAKQADKNANKVRFSDVAGAEEEKQELVEVVEFLKDPRKFSALGARIPAGVLLEGPPGTGKTLLAKAVAGEAGVPFFSISGSDFVEMFVGVGASRVRDLFDQAKKAAPSIIFIDEIDAVGRQRGAGMGGGHDEREQTLNQLLVEMDGFTGNEGVIVIAATNRSDVLDPALLRPGRFDRKILVGRPDVKGREAILKVHAKNKPLAPNVDLKEIARQTPGFVGADLENLLNEAALVAARRSKKQIDAADVDEAEDRVIAGPAKKDRVISPKERTMVAYHEAGHAIVGLVLSDSRTVRKVTIIPRGRAGGYAIMLPKDDQFLLTKKELTEQIVGLLGGRTAEEIIFGVESTGASNDFEQATQIARSMVTQYGMSDRLGTVQLETEGQPFLGAQYGQTPPYSETTATAIDDEVRRIIDEAHKQAYEIIQAHRDQHKLIAEALLKYETLNEKEILSLFNDGKMPERDQDEFPSEKAATFEQSKAALEHKDKEKQADEEAHAKSEGQSNADQTDQDAHDDNTTDSVEKPDDTTPGSNPSDDDHQNPQA

**The amino acid sequence of C2JX39**

MANQIQELTLEEVMGDRFGRYSKYIIQERALPDVRDGLKPVQRRILFAMNQDGNTYDKGFRKSAKSVGNVMGNYHPHGDSSIYEAMVRLSQDWKLRAPLIQMHGNNGSMDGDPPAAMRYTEARLSKISKEMLADIDKETVDMVLNFDDTAQEPTVLPAGFPNLLVNGATGISAGYATEIPPHNLREVVNAILYLLKHPQADLADLMQYVKGPDFPTGGIIQGLDGIKQAYETGRGKIVVRSRTHIETIRGGREKIVVTEIPYDVNKAQMIKKIDELRLNKKVDGIAEVRDESDRFGLSVVIELKKEADAHGILNYLFKNTDLQITYNFNMVAIADMQPKLLGLKAMLEAYVAHRRDVVTRRTRYELNKAQARQHIVEGLIKMLSILDQVIAAIRASSDKGDAKRNLVKQFDFSEAQAEAIVSLQLYRLTNTDITALQDEAAQLAKAIAEYQDILAQPASRDRVIEKELKRIAKEYGDDRRSSIQAEIETLEVATTVTVADETVMVQVSRDGYVKRSSLRSYQAVDPEDNGLKPNDLAIFTGELSTLQHLYIVTNAGNIIYRPVYEISDARWKDTGEHLSQTVGLGTDEKVLAVFAFDQLDLAGTFVLGSSDGYIKQTALSDLQPQRTYKRKPMMAMKLKTPGAVVTNAYFTTDQAQDVFVVSRHAYGLDFPLAEVSTVGARATGVKSMDLKPEDEVVNFILVKQPEKAVVGILTQRGAFKRMALSEVGQMSRARRGLLVLRELKRDPHRIVAMMQVDDNTRLDVLTDADKVITLTPVNHPTGDRYSNGSFVIDTDVQGKPVFVRTREETPVEP

**The amino acid sequence of C2K0J4**

MQGKAEGDQVKATAQAYTKAFANRQYEKAVKQVDTSHLKGPGWQYTAKTLAERNQAVFDRIGASNIKITDLKTTNSKDGTYQLTFTANMNTKIGKLPAQHYTAPIVKVGDNWRIRWTPSLLFPQMDGKDTVQISLTAATRGKILDRNGQALATNGNVTQAGLVPGKLGSGDERTANLAKIATAWDVKTSSLETLLKQSWVTDDTFVPVKIVTDSPALTGAAYQTIGSRTYPLGEAAAQLVGYVGTATADDLKKHPSLTANSKIGKAGLEQIYDHYLRGTDGGTIAIRNGSNSHPLLDTKAVAGKNLKLTIDATKQKTAYTQLAGKSGSVVTMDPTNGELLTLASSPSYDPNAFVNGISQTNYDKYANNTELPFLSRFTQRYASGSTFKMLTAAIALQNKTITPDTTKSISGLKWQKDSSWGDYKVTRTVDAGPENMTQALVNSDNIWFAQVALKMGASAYLKGLEPLFKTQANLPLTMKKAQISNNGKLASETLLADTAYGQGQLLLSPIEQAAMYSAIANDGTMQQPTLIQAAKGKRTSVLQSNAAKTVKTALTHVVSDQAGTAHDLAIDGHTIAAKTGTAELKQKQDTDGKENGFLVAMDADKNTYLTVALIEGTGSGDVVTAMKPFVASLY

* Transmembrane and signal peptide regions of C2JVE6, C2JX39, and C2K0J4 were excised

**Reference**

1. Stals A, Baert L, Botteldoorn N, Werbrouck H, Herman L, Uyttendaele M, Van Coillie E. 2009. Multiplex real-time RT-PCR for simultaneous detection of GI/GII noroviruses and murine norovirus 1. *Journal of Virological Methods* 161:247-253.

2. Phelan PE, Pressley ME, Witten PE, Mellon MT, Blake S, Kim CH. 2005. Characterization of snakehead rhabdovirus infection in zebrafish (*Danio rerio*). *Journal of Virology* 79:1842-1852.
